# Supplementary material for: Exchange coupling torque in ferrimagnetic Co/Gd bilayer maximized near angular momentum compensation temperature
Source: Nat Commun. 2018 Nov 26;9:4984. doi: 10.1038/s41467-018-07373-w (PMC6255835; doi:10.1038/s41467-018-07373-w)
Supplement: Supplementary file 1 — Supplementary Information [file 41467_2018_7373_MOESM1_ESM.pdf]

Supplementary Information for "Exchange coupling torque in ferrimagnetic Co/Gd bilayer maximized near angular momentum compensation temperature"

Bläsing et al.

October 5, 2018

Supplementary Notes

Supplementary Note 1 Temperature dependene of magnetization

1.1 Magnetization of Co/Gd bilayer versus temperature

In Supplementary Figure 1 magnetic hysteresis loops of the Co/Gd bilayer that is discussed in the main text are shown for temperatures ranging from 190 K to 250 K. The magnetization was measured using a Quantum Design Vibrating SQUID magnetometer. The magnetic field is applied perpendicular to the plane of the sample. We note that the magnetization of the sample is small due to the ferrimagnetic alignment of the Co and Gd layers. Approximately around 210 K the magnetization reaches a minimum value indicating the near complete compensation of the Co and Gd magnetization.

1.2 Modelling of temperature dependence of magnetization

We model the temperature dependence by Bloch's law. We assume the temperature dependence of the magnetic moment m_i (= magnetization M_i multiplied thickness t_i) of the i-th's layer is given by a function

$$m_i(T) = m_i^{T=0} \left(1 - \frac{T}{T_i^{\mathrm{C}}}\right)^{\xi_i} \tag{1}$$

where T is the temperature, T_i^{C} is the Curie temperature of layer i and ξ_i is an exponent describing the temperature dependence of layer i. In this SI, we denote the two layers in a more general notation by L for lower and U for upper layer. The lower layer would correspond to the Co and the upper layer to the Gd layer in the bi-layer system of the main text.

At the moment compensation temperature T_{M} the following condition applies

$$\begin{aligned} m_{\mathrm{L}}(T_{\mathrm{M}}) &= m_{\mathrm{U}}(T_{\mathrm{M}}) \\ m_{\mathrm{L}}^{T=0} \left(1 - \frac{T_{\mathrm{M}}}{T_{\mathrm{L}}^{\mathrm{C}}}\right)^{\xi_{\mathrm{L}}} &= m_{\mathrm{U}}^{T=0} \left(1 - \frac{T_{\mathrm{M}}}{T_{\mathrm{U}}^{\mathrm{C}}}\right)^{\xi_{\mathrm{U}}} \\ \kappa^{T=0} &= \left(1 - \frac{T_{\mathrm{M}}}{T_{\mathrm{U}}^{\mathrm{C}}}\right)^{\xi_{\mathrm{U}}} \left(1 - \frac{T_{\mathrm{M}}}{T_{\mathrm{L}}^{\mathrm{C}}}\right)^{-\xi_{\mathrm{L}}} \end{aligned} \tag{2}$$

where $\kappa^{T=0}$ gives the ratio of $m_{\mathrm{L}}^{T=0}$ to $m_{\mathrm{U}}^{T=0}$. Whereas at the total angular momentum compensation temperature T_{A}

$$\begin{aligned} \frac{m_{\mathrm{L}}(T_{\mathrm{A}})}{\gamma_{\mathrm{L}}} &= \frac{m_{\mathrm{U}}(T_{\mathrm{A}})}{\gamma_{\mathrm{U}}} \\ \frac{m_{\mathrm{L}}^{T=0}}{\gamma_{\mathrm{L}}} \left(1 - \frac{T_{\mathrm{A}}}{T_{\mathrm{L}}^{\mathrm{C}}}\right)^{\xi_{\mathrm{L}}} &= \frac{m_{\mathrm{U}}^{T=0}}{\gamma_{\mathrm{U}}} \left(1 - \frac{T_{\mathrm{A}}}{T_{\mathrm{U}}^{\mathrm{C}}}\right)^{\xi_{\mathrm{U}}} \\ \kappa^{T=0} \frac{\gamma_{\mathrm{U}}}{\gamma_{\mathrm{L}}} &= \left(1 - \frac{T_{\mathrm{A}}}{T_{\mathrm{U}}^{\mathrm{C}}}\right)^{\xi_{\mathrm{U}}} \left(1 - \frac{T_{\mathrm{A}}}{T_{\mathrm{L}}^{\mathrm{C}}}\right)^{-\xi_{\mathrm{L}}} . \end{aligned} \tag{3}$$

The experimental temperature dependence of the Co/Gd bi-layer is modelled by Supplementary Equation (1) based on the SQUID data. We assumed that $\kappa^{T=0} = 0.19$ [1] and considered a linear decrease of m_{Co}. We find $T_{\mathrm{Co}}^{\mathrm{C}} = 600$ K (similar to [2]) and $m_{\mathrm{Co}}^{T=0} = 0.7$ mA as well as $m_{\mathrm{Gd}}^{T=0} = 3.6$ mA, $T_{\mathrm{Gd}}^{\mathrm{C}} = 520$ K and $\xi_{\mathrm{Gd}} = 4$. For Gd we note that the exponent of $\xi_{\mathrm{Gd}} = 4$ and the Curie temperature of 520 K are large which describes a rapid decrease of the magnetization with increasing temperature with some small magnetization even above room temperature. The Curie temperature of Gd in this bi-layer is even larger than the bulk value: 293 K [3]. However, this result is in agreement with the observation of magnetization in Gd in a Co/Gd bi-layer at 300 K which was attributed to proximity induced Gd moments at the bi-layer interface [4].

Supplementary Note 2 CIDWM in a AF coupled bi-layer system

We built a model for CIDWM in AF bi-layer which is based on the model for SAF structures proposed in [5, 6]. This is an one-dimensional model for a stripe like nanowire [length (in x direction) $\gg$ width (in y direction) $\gg$ height (in z direction)] with out-of-plane magnetization. We investigate the dynamics in two AF-coupled magnetic layers which are stacked in z direction. Due to the spin Hall effect in the Pt underlayer a spin current is injected into the Co and Gd layer. This creates a STT on the magnetization in each layer and rotates the magnetization out of equilibrium. This gives rise to the exchange coupling torque and DMI torque which move the DWs.

In the model the DW center position is described by q at which the magnetization is in-plane which is expressed by the magnetization's polar angle $\theta|_q = 0$. The rotation of the magnetization in the x-y plane is described by the azimuthal angle ϕ. We will separate the equations for the upper and lower magnetic layer, indexed by U and L. We assume that both are strongly coupled such that the velocity $\dot{q}_{\mathrm{U}} = \dot{q}_{\mathrm{L}} = \dot{q}$.

The domain wall energy is given by:

$$
\begin{aligned}
\hat{\sigma}_i = & 2\left(K_i^{\mathrm{eff}} + K_i^{\mathrm{u}} \cos\phi_i^2\right) t_i\,\Delta_i + 2\,a_i\,t_i\,\frac{1}{\Delta_i} \\
& - \pi\,M_i\,t_i\,(H_x \cos\phi_i + H_y \sin\phi_i)\,\Delta_i \\
& - 2\,J^{\mathrm{ex}} \cos(\phi_i - \phi_j)\,\Delta_i - J^{\mathrm{ex}}\,2\,\Delta_i \\
& \pm \pi\,D_i \cos\phi_i \mp M_i\,t_i\,H_z\,q
\end{aligned}
\tag{4}
$$

with the effective anisotropy K_i^{eff} of layer i, the DW shape anisotropy constant K_i^{u} that favors Bloch walls, the DW width Δ_i, the exchange stiffness a_i, the in-plane fields H_x and H_y, the exchange coupling between both layers J^{ex}, the DMI constant D_i and the out-of-plane field H_z.

Due to the large exchange coupling we make the assumption that $\Delta_{\mathrm{L}} \approx \Delta_{\mathrm{U}}$ and approximate $\Delta_{\mathrm{L}} = \Delta_{\mathrm{U}} = \Delta = \sqrt{\frac{a}{K^{\mathrm{eff}}}}$ where K^{eff} and a are averaged values for the upper and lower layer. As $K^{\mathrm{eff}} = 2\,K^{\mathrm{cryst}}/\,t_{\mathrm{Co+Gd}} - \mu_0\,M_{\mathrm{net}}^2/\,2$ with $M_{\mathrm{net}} = (m_{\mathrm{L}} - m_{\mathrm{U}})/(t_{\mathrm{L}} + t_{\mathrm{U}})$ the DW width varies with temperature. However, due to the low m_{net} the DW width is determined mainly by crystalline anisotropy K^{cryst} and the exchange stiffness a. We assume $K^{\mathrm{cryst}} = 0.7$ J m^{-2} (comparable with [7]) and $a = 0.6 \times 10^{-11}$ J m^{-1} [8] which results in a DW width of about 2 nm. We assume α_{Gd} to be comparable to α_{Co} [9] which we assume to be $\alpha_{\mathrm{Co}} \approx 0.1$ [10].

We use the LLG equation to calculate the DW velocity given by

$$
\frac{\partial \vec{m}_i}{\partial t} = -\gamma\,\vec{m}_i \times \vec{H}_{\mathrm{eff}} + \frac{\alpha}{m_i}\,\vec{m}_i \times \frac{\partial \vec{m}_i}{\partial t} + \vec{\tau},
\tag{5}
$$

We find the following three equations by this.

$$
\begin{aligned}
\dot{q} = & \frac{1}{\frac{M_{\mathrm{L}}\,t_{\mathrm{L}}\,\alpha_{\mathrm{L}}}{\gamma_{\mathrm{L}}\,\Delta_{\mathrm{L}}} + \frac{M_{\mathrm{U}}\,t_{\mathrm{U}}\,\alpha_{\mathrm{U}}}{\gamma_{\mathrm{U}}\,\Delta_{\mathrm{U}}}} \left[\mp\frac{\pi}{2}\frac{\mu_{\mathrm{B}}\,j_{\mathrm{UL}}}{e}\left(\frac{\theta_{\mathrm{L}}^{\mathrm{SH}}}{\gamma_{\mathrm{L}}}\cos\phi_{\mathrm{L}} - \frac{\theta_{\mathrm{U}}^{\mathrm{SH}}}{\gamma_{\mathrm{U}}}\cos\phi_{\mathrm{U}}\right)\right. \\
& \pm (M_{\mathrm{L}}\,t_{\mathrm{L}} - M_{\mathrm{U}}\,t_{\mathrm{U}})\,H_z - \beta_{\mathrm{L}}\frac{\hbar\,P_{\mathrm{L}}\,j_{\mathrm{L}}\,t_{\mathrm{L}}}{e\,\Delta_{\mathrm{L}}} - \beta_{\mathrm{U}}\frac{\hbar\,P_{\mathrm{U}}\,j_{\mathrm{U}}\,t_{\mathrm{U}}}{e\,\Delta_{\mathrm{U}}} \\
& \left.\mp\frac{M_{\mathrm{L}}\,t_{\mathrm{L}}}{\gamma_{\mathrm{L}}}\dot{\phi}_{\mathrm{L}} \pm \frac{M_{\mathrm{U}}\,t_{\mathrm{U}}}{\gamma_{\mathrm{U}}}\dot{\phi}_{\mathrm{U}}\right]
\end{aligned}
\tag{6}
$$

$$
\begin{aligned}
\dot{\phi}_{\mathrm{L}} = & \pm\frac{1}{\alpha_{\mathrm{L}}\Delta_{\mathrm{L}}}\dot{q} + \frac{\gamma_{\mathrm{L}}}{\alpha_{\mathrm{L}}}\left[\frac{H_{\mathrm{L}}^{\mathrm{d}}}{2}\sin 2\phi_{\mathrm{L}} \pm \frac{\pi}{2}\frac{D_{\mathrm{L}}}{\Delta_{\mathrm{L}}\,M_{\mathrm{L}}\,t_{\mathrm{L}}}\sin\phi_{\mathrm{L}}\right. \\
& \left.-\frac{J^{\mathrm{ex}}}{M_{\mathrm{L}}\,t_{\mathrm{L}}}\sin(\phi_{\mathrm{L}} - \phi_{\mathrm{U}}) - \frac{\pi}{2}(H_x\sin\phi_{\mathrm{L}} - H_y\cos\phi_{\mathrm{L}}) \pm \frac{\hbar P_{\mathrm{L}} j_{\mathrm{L}}}{e M_{\mathrm{L}}\Delta_{\mathrm{L}}}\right]
\end{aligned}
\tag{7}
$$

$$
\begin{aligned}
\dot{\phi}_{\mathrm{U}} = & \mp\frac{1}{\alpha_{\mathrm{U}}\Delta_{\mathrm{U}}}\dot{q} + \frac{\gamma_{\mathrm{U}}}{\alpha_{\mathrm{U}}}\left[\frac{H_{\mathrm{U}}^{\mathrm{d}}}{2}\sin 2\phi_{\mathrm{U}} \mp \frac{\pi}{2}\frac{D_{\mathrm{U}}}{\Delta_{\mathrm{U}}\,M_{\mathrm{U}}\,t_{\mathrm{U}}}\sin\phi_{\mathrm{U}}\right. \\
& \left.-\frac{J^{\mathrm{ex}}}{M_{\mathrm{U}}\,t_{\mathrm{U}}}\sin(\phi_{\mathrm{U}} - \phi_{\mathrm{L}}) - \frac{\pi}{2}(H_x\sin\phi_{\mathrm{U}} - H_y\cos\phi_{\mathrm{U}}) \mp \frac{\hbar P_{\mathrm{U}} j_{\mathrm{U}}}{e M_{\mathrm{U}}\Delta_{\mathrm{U}}}\right]
\end{aligned}
\tag{8}
$$

θ_i^{SH} is the effective spin Hall angle which is the spin current in layer i contributing to the STT as a fraction of the conventional current flowing through the Pt underlayer. β_i is the non-adiabatic STT coefficient and P_i the spin

polarization of the current in layer i. This spin current is polarized in $-y$ or $+y$ direction for a current density j_{UL} in the underlayer in $+x$ or $-x$ direction, respectively. D_i is the DMI constant within each layer. In order to fit our data, we have to use $D_{\mathrm{Co}} = 0.2$ pJ m^{-1}. This is small but within the range reported for a Co-Tb alloy [11]. Furthermore, note that K_i^{u} is small here as at magnetization compensation point the demagnetization field H_i^{d} disappears.

$\dot{\phi}_{\mathrm{U}}$ and $\dot{\phi}_{\mathrm{L}}$ can be inserted in Supplementary Equation (6) such that

$$\begin{aligned}\dot{q} =& \frac{1}{\frac{M_{\mathrm{L}}\,t_{\mathrm{L}}(1+\alpha_{\mathrm{L}}^2)}{\gamma_{\mathrm{L}}\,\Delta_{\mathrm{L}}\,\alpha_{\mathrm{L}}} + \frac{M_{\mathrm{U}}\,t_{\mathrm{U}}(1+\alpha_{\mathrm{U}}^2)}{\gamma_{\mathrm{U}}\,\Delta_{\mathrm{U}}\,\alpha_{\mathrm{U}}}} \\ & * \left\{ \mp \frac{M_{\mathrm{L}}\,t_{\mathrm{L}}}{\alpha_L} \left[\frac{H_{\mathrm{L}}^{\mathrm{d}}}{2} \sin 2\phi_{\mathrm{L}} \pm \frac{\pi}{2} \frac{D_{\mathrm{L}}}{\Delta_{\mathrm{L}}\,M_{\mathrm{L}}\,t_{\mathrm{L}}} \sin\phi_{\mathrm{L}} - \frac{J^{\mathrm{ex}}}{M_{\mathrm{L}}\,t_{\mathrm{L}}} \sin(\phi_{\mathrm{L}} - \phi_{\mathrm{U}}) \right.\right. \\ & - \frac{\pi}{2}(H_x \sin\phi_{\mathrm{L}} - H_y \cos\phi_{\mathrm{L}}) + \frac{\pi}{2}\alpha_{\mathrm{L}} \frac{\mu_{\mathrm{B}}\,\theta_{\mathrm{L}}^{\mathrm{SH}}\,J_{\mathrm{UL}}}{\gamma_{\mathrm{L}}\,e\,M_{\mathrm{L}} t_{\mathrm{L}}} \cos\phi_{\mathrm{L}} - \alpha_{\mathrm{L}} H_z \\ & \left. \pm (1+\alpha_{\mathrm{L}}\beta_{\mathrm{L}}) \frac{\hbar P_{\mathrm{L}} J_{\mathrm{L}}}{e M_{\mathrm{L}} \Delta_{\mathrm{L}}} \right] \\ & \pm \frac{M_{\mathrm{U}}\,t_{\mathrm{U}}}{\alpha_{\mathrm{U}}} \left[\frac{H_{\mathrm{U}}^{\mathrm{d}}}{2} \sin 2\phi_{\mathrm{U}} \mp \frac{\pi}{2} \frac{D_{\mathrm{U}}}{\Delta_{\mathrm{U}} M_{\mathrm{U}}\,t_{\mathrm{U}}} \sin\phi_{\mathrm{U}} - \frac{J^{\mathrm{ex}}}{M_{\mathrm{U}}\,t_{\mathrm{U}}} \sin(\phi_{\mathrm{U}} - \phi_{\mathrm{L}}) \right. \\ & - \frac{\pi}{2}(H_x \sin\phi_{\mathrm{U}} - H_y \cos\phi_{\mathrm{U}}) + \frac{\pi}{2}\alpha_{\mathrm{U}} \frac{\mu_{\mathrm{B}}\,\theta_{\mathrm{U}}^{\mathrm{SH}}\,J_{\mathrm{UL}}}{\gamma_{\mathrm{U}}\,e\,M_{\mathrm{U}}\,t_{\mathrm{U}}} \cos\phi_{\mathrm{U}} - \alpha_{\mathrm{U}} H_z \\ & \left.\left. \mp (1+\alpha_{\mathrm{U}}\beta_{\mathrm{U}}) \frac{\hbar P_{\mathrm{U}} J_{\mathrm{U}}}{e M_{\mathrm{U}} \Delta_{\mathrm{U}}} \right] \right\}\end{aligned} \tag{9}$$

In this form the velocity can be split into the contributions of each torque to the total final velocity.

In the limit of extremely large exchange coupling ($J^{\mathrm{SH}} \ll J^{\mathrm{ex}}$ and $J^{\mathrm{DMI}} \ll J^{\mathrm{ex}}$), $\phi_{\mathrm{L}} \approx \phi_{\mathrm{U}} + \pi$ and $\delta\phi \ll 1$. The equations of motion can then be rewritten

Supplementary Note 3 Steady state motion

For steady state motion $\dot{\phi}_{\mathrm{U}} = \dot{\phi}_{\mathrm{L}} = 0$. Hence, Supplementary Equations (6), (7) and (8) can be written as follows.

Steady state of Supplementary Equation (6):

$$\begin{aligned}\dot{q} =& \frac{1}{\frac{M_{\mathrm{L}}\,t_{\mathrm{L}}\,\alpha_{\mathrm{L}}}{\gamma_{\mathrm{L}}\,\Delta_{\mathrm{L}}} + \frac{M_{\mathrm{U}}\,t_{\mathrm{U}}\,\alpha_{\mathrm{U}}}{\gamma_{\mathrm{U}}\,\Delta_{\mathrm{U}}}} \left[\mp\frac{\pi}{2} M_{\mathrm{L}}\,t_{\mathrm{L}}\,H_{\mathrm{L}}^{\mathrm{SH}}\,\cos\phi_{\mathrm{L}} \pm \frac{\pi}{2} M_{\mathrm{U}}\,t_{\mathrm{U}}\,H_{\mathrm{U}}^{\mathrm{SH}}\,\cos\phi_{\mathrm{U}} \right. \\ & \pm (M_{\mathrm{L}}\,t_{\mathrm{L}} - M_{\mathrm{U}}\,t_{\mathrm{U}})\,H_z - \beta_{\mathrm{L}} \frac{M_{\mathrm{L}}\,t_{\mathrm{L}}}{\Delta_{\mathrm{L}}\,\gamma_{\mathrm{L}}} u_{\mathrm{L}} - \beta_{\mathrm{U}} \frac{M_{\mathrm{U}}\,t_{\mathrm{U}}}{\Delta_{\mathrm{U}}\,\gamma_{\mathrm{U}}} u_{\mathrm{U}} \\ & \left. \underbrace{\mp \frac{M_{\mathrm{L}}\,t_{\mathrm{L}}}{\gamma_{\mathrm{L}}} \dot{\phi}_{\mathrm{L}} \pm \frac{M_{\mathrm{U}}\,t_{\mathrm{U}}}{\gamma_{\mathrm{U}}} \dot{\phi}_{\mathrm{U}}}_{=0} \right]\end{aligned} \tag{10}$$

Steady state of Supplementary Equation (7):

$$\begin{aligned}\dot{q} =& \mp \frac{\alpha_{\mathrm{L}}}{\frac{M_{\mathrm{L}}\,t_{\mathrm{L}}\,\alpha_{\mathrm{L}}}{\gamma_{\mathrm{L}}\,\Delta_{\mathrm{L}}}} \left[\pm\frac{\pi}{2} M_{\mathrm{L}}\,t_{\mathrm{L}}\,H_{\mathrm{L}}^{\mathrm{DMI}} \sin\phi_{\mathrm{L}} - M_{\mathrm{L}}\,t_{\mathrm{L}} \frac{H_{\mathrm{L}}^{\mathrm{ex}}}{2} \sin(\phi_{\mathrm{L}} - \phi_{\mathrm{U}}) \right. \\ & + M_{\mathrm{L}}\,t_{\mathrm{L}} \frac{H_{\mathrm{L}}^{\mathrm{d}}}{2} \sin 2\phi_{\mathrm{L}} - \frac{\pi}{2} M_{\mathrm{L}}\,t_{\mathrm{L}}\,(H_x \sin\phi_{\mathrm{L}} - H_y \cos\phi_{\mathrm{L}}) \pm \frac{M_{\mathrm{L}}\,t_{\mathrm{L}}}{\Delta_{\mathrm{L}}\,\gamma_{\mathrm{L}}} u_{\mathrm{L}} \\ & \left. \underbrace{- \frac{\alpha_{\mathrm{L}}\,M_{\mathrm{L}}\,t_{\mathrm{L}}}{\gamma_{\mathrm{L}}} \dot{\phi}_{\mathrm{L}}}_{=0} \right]\end{aligned} \tag{11}$$

Steady state of Supplementary Equation (8):

$$\begin{aligned}\dot{q} = \pm \frac{\alpha_{\rm U}}{\frac{M_{\rm U}\, t_{\rm U}\, \alpha_{\rm U}}{\gamma_{\rm U}\, \Delta_{\rm U}}} \Big[&\mp\frac{\pi}{2} M_{\rm U}\, t_{\rm U}\, H_{\rm U}^{\rm DMI} \sin\phi_{\rm U} - M_{\rm U}\, t_{\rm U} \frac{H_{\rm U}^{\rm ex}}{2} \sin\left(\phi_{\rm U} - \phi_{\rm L}\right) \\ &+ M_{\rm U}\, t_{\rm U} \frac{H_{\rm U}^{\rm d}}{2} \sin 2\phi_{\rm U} - \frac{\pi}{2} M_{\rm U}\, t_{\rm U}\, \left(H_x \sin\phi_{\rm U} - H_y \cos\phi_{\rm U}\right) \mp \frac{M_{\rm U}\, t_{\rm U}}{\Delta_{\rm U}\, \gamma_{\rm U}} u_{\rm U} \\ &\underbrace{- \frac{\alpha_{\rm U}\, M_{\rm U}\, t_{\rm U}}{\gamma_{\rm U}} \dot{\phi}_{\rm U}}_{=0} \Big]\end{aligned} \tag{12}$$

with $H_i^{\rm SH} = \frac{\mu_{\rm B}\, \theta_i^{\rm SH}\, j_{\rm UL}}{\gamma_i\, e\, M_i\, t_i}$ and $u_i = \frac{\gamma_i\, \hbar\, P_i\, j_i}{e\, M_i}$, $H_i^{\rm ex} = \frac{2\, J^{\rm ex}}{M_i\, t_i}$ and $H_i^{\rm DMI} = \frac{D_i}{\Delta_i\, M_i\, t_i}$.

Supplementary Note 4 Pinning in steady state motion

In our experiments we find that a threshold current is needed to initiate DW motion which we attribute to pinning from defects. Thus, we consider the case of steady state DW motion in the presence of a pinning field $H_i^{\rm pinning} = K_i^{\rm df}\, M_i\, t_i$ where $K_i^{\rm df}$ is a constant which accounts for dry friction (cf. [12]). The dry friction can be associated with the torque term:

$$\tau_i^{\rm df} = \gamma\, H_i^{\rm pinning} \left(\mathbf{m}_i \times \frac{\frac{\partial \mathbf{m}_i}{\partial t}}{\left|\frac{\partial \mathbf{m}_i}{\partial t}\right|} \right). \tag{13}$$

The resulting steady state velocity (Supplementary Equation (10)) is then given by:

$$\begin{aligned}\dot{q} = \frac{1}{\frac{M_{\rm L}\, t_{\rm L}\, \alpha_{\rm L}}{\gamma_{\rm L}\, \Delta_{\rm L}} + \frac{M_{\rm U}\, t_{\rm U}\, \alpha_{\rm U}}{\gamma_{\rm U}\, \Delta_{\rm U}}} \Big[&\mp\frac{\pi}{2} M_{\rm L}\, t_{\rm L}\, H_{\rm L}^{\rm SH} \cos\phi_{\rm L} \pm \frac{\pi}{2} M_{\rm U}\, t_{\rm U}\, H_{\rm U}^{\rm SH} \cos\phi_{\rm U} \\ &\pm \left(M_{\rm L}\, t_{\rm L} - M_{\rm U}\, t_{\rm U}\right) H_z - \beta_{\rm L} \frac{M_{\rm L}\, t_{\rm L}}{\Delta_{\rm L}\, \gamma_{\rm L}} u_{\rm L} - \beta_{\rm U} \frac{M_{\rm U}\, t_{\rm U}}{\Delta_{\rm U}\, \gamma_{\rm U}} u_{\rm U} \\ &- \frac{\pi}{2} \left(M_{\rm L}\, t_{\rm L}\, H_{\rm L}^{\rm pinning} + M_{\rm U}\, t_{\rm U}\, H_{\rm U}^{\rm pinning}\right) \Big].\end{aligned} \tag{14}$$

As the main focus of this paper is the exchange coupling torque, we simply adjust $K_i^{\rm df}$ such that it accounts for the experimentally observed threshold current. We find that $K_{\rm Co}^{\rm df} = 50$ T A^{-1} and $K_{\rm Gd}^{\rm df} = 0$. We note that the detailed origin of the pinning is not clear but this model is able to describe both the j and H_x dependence of the DW velocity well.

Supplementary Note 5 H_x dependence at $T_{\rm A}$

The temperature $T^{\zeta=0}$, at which H_x has no effect on the DW velocity, depends on the torque created by H_x on the magnetization. If the torque provided by H_x ($\tau_i^{H_x} = -\gamma\, \mathbf{m}_i \times \mathbf{H}_x$) in the upper and lower layers cancels out, there would then be no effect of H_x on the DW velocity. In order that the torque cancels out, $m_{\rm L}$ and $m_{\rm U}$ have to be aligned towards the same side with respect to H_x (both towards $+y$ or both towards -y). This is possible because the spin torque arising from the spin current from the underlayer, rotates both $m_{\rm L}$ and $m_{\rm U}$ into the same direction resulting in $\phi_{\rm L} - \phi_{\rm U} \neq \pi$. Thus, we need to find out over which temperatures range $\phi_{\rm L}$ and $\phi_{\rm U}$ are pointing in the same y direction. We use $\zeta_i = \frac{M_i\, t_i\, \alpha_i}{\gamma_i\, \Delta_i}$ with $[\zeta_i] =$ J s m^{-3}, $J_i^{\rm SH} = \frac{\pi}{2} \frac{\mu_{\rm B}\, \theta_i^{\rm SH}\, j_{\rm UL}}{\gamma_i\, e}$ with $[J_i^{\rm SH}] =$ J m^{-2} and $J_i^{\rm DMI} = \frac{\pi}{2} \frac{D_i}{\Delta_i}$ with $[J_i^{\rm DMI}] =$ J m^{-2}. For the discussion in this section we neglect the effect of pinning.

For the lower bound ($= T^{\rm lower}$), we find that $\phi_{\rm L}$ is always π for a ↑↓ DW (or 0 for ↓↑ DW), as will be discussed in Supplementary Note 8 in detail, at the angular compensation point ($\Rightarrow T^{\rm lower} = T_{\rm A}$) where

$$\frac{m_{\rm L}}{\gamma_{\rm L}} = \frac{m_{\rm U}}{\gamma_{\rm U}}. \tag{15}$$

With $g_{\rm Co} = 2.2$ and $g_{\rm Gd} = 2.0$ this corresponds to $m_{\rm Co} = 0.91 \times m_{\rm Gd}$. For positive current densities, below $T^{\rm lower}$, $\phi_{\rm Co}$ is always in the $+y$ $(-y)$ direction for a ↑↓ (↓↑) DW, and $\phi_{\rm Gd}$ is always along $-y$ $(+y)$, accordingly. Just

above T^{lower}, ϕ_{Co} is also along $-y$ $(+y)$ while the direction of ϕ_{Gd} in respect to y changes at a higher temperature T^{upper}. Note that for negative current densities the rotation into the $+y$ or $-y$ directions is reversed.

To determine T^{upper} at which ϕ_{Gd} changes its direction in respect to y, we solve Supplementary Equations (11) and (12) for $\phi_{\text{Gd}} = 0$ for a $\uparrow\downarrow$ DW (or π for $\downarrow\uparrow$ DW). This gives

$$\frac{m_{\text{L}}}{\gamma_{\text{L}}} = \frac{m_{\text{U}}}{\gamma_{\text{U}}}\left(1 - \frac{J_{\text{L}}^{\text{DMI}}}{J^{\text{ex}}}\right) \tag{16}$$

which corresponds to $m_{\text{Co}} = 0.91 \times m_{\text{Gd}} \times (1 - J_{\text{L}}^{\text{DMI}}/J^{\text{ex}})$. Just below T^{upper}, ϕ_{Gd} is always at the $-y$ $(+y)$ side like ϕ_{Co}. Above T^{upper}, ϕ_{Gd} is on the $+y$ $(-y)$ side while ϕ_{Co} is on the $-y$ $(+y)$ side. Note that for negative current densities the rotation into the $+y$ or $-y$ directions is reversed.

T^{lower} and T^{upper} define the temperature range where we can expect $\dot{q}$ to be independent of H_x. If we assume that $J_{\text{L}}^{\text{DMI}}/J^{\text{ex}}$ is small, these two temperatures become very close. In our calculations we use $J_{\text{L}}^{\text{DMI}} = \pi/2 \times 0.2$ pJ m^{-1} / 2 nm = 0.16 mJ m^{-2} and $J^{\text{ex}} = -0.9$ mJ m^{-2}. This results in $J_{\text{L}}^{\text{DMI}}/J^{\text{ex}} \approx 0.17$. To find the exact temperature $T^{\zeta=0}$ we now explore the formulas in more detail.

We are looking for $\frac{d\dot{q}(H_x)}{dH_x} = 0$. Thus, we start from the fact that in for steady state motion the angles ϕ_{L} and ϕ_{U} are determined by all in-plane fields including the exchange coupling, DMI field and especially H_x (cf. Supplementary Equations (11) and (12)). This means $\phi_{\text{L}}(H_x)$ and $\phi_{\text{U}}(H_x)$. From Supplementary Equation (10) we know that the changes of ϕ_{L} and ϕ_{U} directly influence the DW velocity $\dot{q}(\phi_{\text{L}}, \phi_{\text{U}})$. Thus, we can write $\frac{d\dot{q}}{dH_x} = \frac{\partial \dot{q}}{\partial \phi_{\text{L}}}\frac{\partial \phi_{\text{L}}}{\partial H_x} + \frac{\partial \dot{q}}{\partial \phi_{\text{U}}}\frac{\partial \phi_{\text{U}}}{\partial H_x}$.

We define the difference

$$\delta\phi = \phi_{\text{L}} - \phi_{\text{U}} - \pi \tag{17}$$

which also takes the AF coupling into account. Due to Supplementary Equation (17) we can write

$$\frac{d\dot{q}}{dH_x} = \begin{cases} \frac{\partial \dot{q}}{\partial \phi_{\text{L}}}\frac{\partial \phi_{\text{L}}}{\partial H_x} + \frac{\partial \dot{q}}{\partial \phi_{\text{L}}}\frac{\partial \phi_{\text{L}}}{\partial \phi_{\text{U}}}\frac{\partial \phi_{\text{U}}}{\partial H_x} = \frac{\partial \dot{q}}{\partial \phi_{\text{L}}}\left(\frac{\partial \phi_{\text{L}}}{\partial H_x} + \frac{\partial \phi_{\text{L}}}{\partial \phi_{\text{U}}}\frac{\partial \phi_{\text{U}}}{\partial H_x}\right) \\ \frac{\partial \dot{q}}{\partial \phi_{\text{U}}}\frac{\partial \phi_{\text{U}}}{\partial H_x} + \frac{\partial \dot{q}}{\partial \phi_{\text{U}}}\frac{\partial \phi_{\text{U}}}{\partial \phi_{\text{L}}}\frac{\partial \phi_{\text{L}}}{\partial H_x} = \frac{\partial \dot{q}}{\partial \phi_{\text{U}}}\left(\frac{\partial \phi_{\text{U}}}{\partial H_x} + \frac{\partial \phi_{\text{U}}}{\partial \phi_{\text{L}}}\frac{\partial \phi_{\text{L}}}{\partial H_x}\right) \end{cases} \tag{18}$$

and aim to find

$$\frac{\partial \dot{q}}{\partial \phi_{\substack{\text{U}\\\text{L}}}} = 0. \tag{19}$$

We substitute ϕ_{U} or ϕ_{L} in Supplementary Equation (10) by Supplementary Equation (17). Furthermore, we approximate $\cos\phi|_{\phi_{\substack{\text{U}\\\text{L}}}=0} \approx 1 - \frac{\phi^2_{\substack{\text{U}\\\text{L}}}}{2}$. Note that the notation $\phi_{\substack{\text{U}\\\text{L}}}$ is consistent with signs for $\uparrow\downarrow$ and $\downarrow\uparrow$ DWs where the upper label is for a $\uparrow\downarrow$ and the lower for a $\downarrow\uparrow$ DW. ϕ_{L} and ϕ_{U} will be around π or 0 for a $\uparrow\downarrow$ and around 0 and π for a $\downarrow\uparrow$ DW, respectively. Thus

$$\dot{q} = \begin{cases} \frac{1}{\zeta_{\text{L}}+\zeta_{\text{U}}}\left[J_{\text{L}}^{\text{SH}}\left(1 - \frac{(\phi_{\text{U}}+\delta\phi)^2}{2}\right) + J_{\text{U}}^{\text{SH}}\left(1 - \frac{\phi_{\text{U}}{}^2}{2}\right)\right] & \text{for } \uparrow\downarrow \text{ DW} \\ \frac{1}{\zeta_{\text{L}}+\zeta_{\text{U}}}\left[J_{\text{L}}^{\text{SH}}\left(1 - \frac{\phi_{\text{L}}{}^2}{2}\right) + J_{\text{U}}^{\text{SH}}\left(1 - \frac{(\phi_{\text{L}}-\delta\phi)^2}{2}\right)\right] & \text{for } \downarrow\uparrow \text{ DW.} \end{cases} \tag{20}$$

As discussed above, we want to find the angles at which there is the least influence on the velocity by any change of ϕ_{U} or ϕ_{L}. This is where $\frac{d\dot{q}}{d\phi_{\substack{\text{U}\\\text{L}}}} = 0$. With

$$\frac{d\dot{q}}{d\phi_{\substack{\text{U}\\\text{L}}}} = \begin{cases} \frac{-1}{\zeta_{\text{L}}+\zeta_{\text{U}}}\left[J_{\text{L}}^{\text{SH}}\left(\phi_{\text{U}} + \delta\phi\right) + J_{\text{U}}^{\text{SH}}\phi_{\text{U}}\right] & \text{for } \uparrow\downarrow \text{ DW} \\ \frac{-1}{\zeta_{\text{L}}+\zeta_{\text{U}}}\left[J_{\text{L}}^{\text{SH}}\phi_{\text{L}} + J_{\text{U}}^{\text{SH}}\left(\phi_{\text{L}} - \delta\phi\right)\right] & \text{for } \downarrow\uparrow \text{ DW} \end{cases} \tag{21}$$

we find

$$\frac{d\dot{q}}{d\phi_{\substack{\text{U}\\\text{L}}}} = 0 \Rightarrow \begin{cases} \phi_{\text{L}}^* = \frac{J_{\text{L}}^{\text{SH}}}{J_{\text{L}}^{\text{SH}}+J_{\text{U}}^{\text{SH}}}\delta\phi - \pi,\ \phi_{\text{U}}^* = \frac{-J_{\text{U}}^{\text{SH}}}{J_{\text{L}}^{\text{SH}}+J_{\text{U}}^{\text{SH}}}\delta\phi & \text{for } \uparrow\downarrow \text{ DW} \\ \phi_{\text{L}}^* = \frac{J_{\text{L}}^{\text{SH}}}{J_{\text{L}}^{\text{SH}}+J_{\text{U}}^{\text{SH}}}\delta\phi,\ \phi_{\text{U}}^* = \frac{-J_{\text{U}}^{\text{SH}}}{J_{\text{L}}^{\text{SH}}+J_{\text{U}}^{\text{SH}}}\delta\phi + \pi & \text{for } \downarrow\uparrow \text{ DW.} \end{cases} \tag{22}$$

Now, we want to find the corresponding ratio of $m_\mathrm{L}/m_\mathrm{U}$ where this is satisfied. Hence, ϕ^*_L and ϕ^*_U are inserted in Supplementary Equations (11) and (12):

$$\dot{q} = \mp \frac{1}{\frac{m_\mathrm{L}}{\gamma_\mathrm{L}}\Delta} \left[\left(\pm J^\mathrm{DMI}_\mathrm{L} - J^{H_x}_\mathrm{L} \right) \sin\phi^*_\mathrm{L} - J^\mathrm{ex} \sin\left(\phi^*_\mathrm{L} - \phi^*_\mathrm{U}\right) \right] \tag{23}$$

$$\dot{q} = \pm \frac{1}{\frac{m_\mathrm{U}}{\gamma_\mathrm{U}}\Delta} \left[\left(\mp J^\mathrm{DMI}_\mathrm{U} - J^{H_x}_\mathrm{U} \right) \sin\phi^*_\mathrm{U} + J^\mathrm{ex} \sin\left(\phi^*_\mathrm{L} - \phi^*_\mathrm{U}\right) \right] \tag{24}$$

We assume that $\delta\phi$ is small such that $\sin\delta\phi \approx \delta\phi$ which results in

$$\dot{q} = \mp \frac{1}{\frac{m_\mathrm{L}}{\gamma_\mathrm{L}}\Delta} \left[\left(-J^\mathrm{DMI}_\mathrm{L} \pm J^{H_x}_\mathrm{L} \right) \frac{J^\mathrm{SH}_\mathrm{L}}{J^\mathrm{SH}_\mathrm{L} + J^\mathrm{SH}_\mathrm{U}} \delta\phi + J^\mathrm{ex}\delta\phi \right] \tag{25}$$

$$\dot{q} = \pm \frac{1}{\frac{m_\mathrm{U}}{\gamma_\mathrm{U}}\Delta} \left[\left(J^\mathrm{DMI}_\mathrm{U} \pm J^{H_x}_\mathrm{U} \right) \frac{J^\mathrm{SH}_\mathrm{U}}{J^\mathrm{SH}_\mathrm{L} + J^\mathrm{SH}_\mathrm{U}} \delta\phi - J^\mathrm{ex}\delta\phi \right] \tag{26}$$

with $J^{H_x}_i = \frac{\pi}{2} m_i H_x$. Now we equate both equations which leads to the general form

$$\frac{m_\mathrm{L}}{\gamma_\mathrm{L}} = \frac{m_\mathrm{U}}{\gamma_\mathrm{U}} \left[\frac{\left(-J^\mathrm{DMI}_\mathrm{L} \pm J^{H_x}_\mathrm{L} \right) + (1+\eta) J^\mathrm{ex}}{\eta \left(-J^\mathrm{DMI}_\mathrm{U} \mp J^{H_x}_\mathrm{U} \right) + (1+\eta) J^\mathrm{ex}} \right]. \tag{27}$$

where $\eta = \frac{\theta^\mathrm{SH}_\mathrm{L}}{\theta^\mathrm{SH}_\mathrm{U}} \frac{\gamma_\mathrm{U}}{\gamma_\mathrm{L}}$. For H_x around 0 and the same assumptions as above ($J^\mathrm{DMI}_\mathrm{U} = 0$), this results in the final equation

$$\frac{m_\mathrm{L}}{\gamma_\mathrm{L}} = \frac{m_\mathrm{U}}{\gamma_\mathrm{U}} \left(1 - \frac{J^\mathrm{DMI}_\mathrm{L}}{J^\mathrm{ex}} \frac{1}{\eta + 1} \right). \tag{28}$$

This point is within the upper and lower bound (Supplementary Equations (15) and (16)) and describes the temperature $T^{\zeta=0}$. If the spin Hall current only acts on the lower layer, there is no influence of H_x on the velocity exactly at the angular momentum compensation (T^lower). If the spin Hall current only acts in the upper layer, there is no influence of H_x on the velocity where Supplementary Equation (16) is satisfied (T^upper).

Note that this result can become current density dependent for very large current densities because the approximation $\sin\delta\phi \approx \delta\phi$ might not be valid in this case. However, we find from our numerical solutions that for current densities below 3×10^8 A cm^{-2} this result still holds reasonably well.

Supplementary Note 6 Numerical modeling of Joule heating in racetrack

In this section, we describe the finite element model used to estimate the overall heating in our device and also present a series of simulation data that explains the experimentally observed heating effects in the current driven domain wall motion of magnetic Co/Gd bilayers. We use the Joule heating module of the commercial software COMSOL Multiphysics and solve for the temperature increase in the device. As shown in Supplementary Figure 10a, a rectangular voltage pulse of amplitude V_0 and pulse-duration of t_p is applied at one leg of the racetrack while keeping the other end at a ground potential. The resulting current density j causes Joule-heating which is mostly concentrated at the narrower section of the device. We aim to find the time t evolution of the temperature T by numerically solving the transient heat-diffusion equation:

$$\rho C_p \frac{\partial T}{\partial t} = \kappa \nabla^2 T + Q_\mathrm{e},$$

where ρ is the density, C_p is the specific heat capacity, κ is the thermal conductivity, $Q_\mathrm{e} = j^2/\sigma$ is the heat generated by Joule-heating which is proportional to the current density j. As the measurements are mostly performed below room temperature and in a vacuum, we do not consider convective and radiative heat losses as they contribute only to a maximum of 5 % temperature (confirmed via an independent control simulation) change towards the the heat diffusion processes. It is straightforward to incorporate such loses in the model by setting outward heat flux boundary proportional to the device temperature rise and heat-transfer coefficient of the metal-vacuum interface.

6.1 Material properties

This three-dimensional heat diffusion equation is solved (in the time-domain) for a device-geometry shown in Supplementary Figure 10a with carefully chosen input parameters, such as the electrical conductivity σ, thermal conductivity κ, specific heat capacity C_p and density ρ. The temperature of the device is initially set to the base temperature of the cryostat and the δT is calculated in the time interval $t = [0, 2t_\mathrm{p}]$, where t_p is the pulse duration, with a time-step of 0.2 ns. How fast the temperature rises depends on the initial temperature of the sample and thermal diffusivity $\alpha = \kappa/(\rho C_\mathrm{p})$ of the materials.

The device modelled here is comprised of a stack sequence of Si / 250 SiOx / 100 AlOx / 20 TaN / 30 Pt / 5 Co / 18 Gd / 50 TaN (All thicknesses in Å). We model the active part of the racetrack (30 Pt / 5 Co / 18 Gd / 50 TaN) as a single metallic film whose electrical conductivity σ is obtained from a separate measurement of the racetrack resistance as a function of temperature (c.f. Supplementary Figure 7). Because heat transport in metals is largely dominated by the electronic contribution (but not by the phonons), it suffices to estimate the thermal conductivity κ of the racetrack using the well-known Wiedemann-Franz relation as $\kappa = L_0 \sigma T_0$, where $L_0 = 2.44 \times 10^{-8}\ \mathrm{V^2K^{-2}}$ is the Lorenz number and T_0 is the reference temperature. We set the specific heat capacity C_p and densities ρ equal to the average of the layers in the racetrack. Because the thermal properties of SiO_2, TaN and AlO_x are very close to each other, the remaining component of the stack (250 SiOx / 100 AlOx / 20 TaN) is modeled as a 370 Å thick SiO_2 substrate with an effective thermal conductivity of 1.5W/(mK)[13]. Heat diffusion to the bottom of the Si substrate is also included by setting the bottom surface of the 500 μm thick Si wafer to the base temperature of the cryostat.

6.2 Results

Supplementary Figure 10a shows a representative temperature profile of a device obtained from the three-dimensional model. Supplementary Figure 10b shows the average volume temperature of the narrow section of the device as a function of time for a 10 ns voltage pulse. Different curves are for various values of j at $T = 210\mathrm{K}$. As shown in Supplementary Figure 10c,the temperature-rise δT is quadratic in j where the maximum temperature for each curve in Supplementary Figure 10b is plotted as a function of j.

For a similar temperature and j, the pulse duration t_p dependence of δT is shown in Supplementary Figure 10d. In the experiment, the estimated temperature rise is obtained after some time-delay and thus does not represent the maximum heating in the device. To make a good comparison with our model, we present the time-averaged δT of the racetrack microwire as shown in red open square symbols, which agree well within 10% of the measurements (shown in blue circle). Given that most of the input-material parameters are obtained from the literature and only the electrical conductivity of the racetrack was measured, an agreement of the simulation data to within 10% of the measured δT indicates that the modeling procedure we followed here captures the main heat diffusion/transport processes occurring in the current-driven motion of domain walls in racetracks.

6.3 DW dynamics for time-varying moments

We now consider the effect of the time-dependent heating during the current pulses of length t_p on the DW dynamics. From Supplementary Equation (6) with $H_z = 0$ and $\beta_i = 0$ for simplicity we find:

$$\begin{aligned}\dot{q} =& \frac{1}{\frac{M_\mathrm{L}\, t_\mathrm{L}\, \alpha_\mathrm{L}}{\gamma_\mathrm{L}\, \Delta_\mathrm{L}} + \frac{M_\mathrm{U}\, t_\mathrm{U}\, \alpha_\mathrm{U}}{\gamma_\mathrm{U}\, \Delta_\mathrm{U}}} \left[\mp \frac{\pi}{2} \frac{\mu_\mathrm{B}\, j_\mathrm{UL}}{e} \left(\frac{\theta_\mathrm{L}^\mathrm{SH}}{\gamma_\mathrm{L}} \cos\phi_\mathrm{L} - \frac{\theta_\mathrm{U}^\mathrm{SH}}{\gamma_\mathrm{U}} \cos\phi_\mathrm{U} \right) \right. \\ & \left. \mp \frac{M_\mathrm{L}\, t_\mathrm{L}}{\gamma_\mathrm{L}} \dot{\phi}_\mathrm{L} \pm \frac{M_\mathrm{U}\, t_\mathrm{U}}{\gamma_\mathrm{U}} \dot{\phi}_\mathrm{U} \right]\end{aligned} \tag{29}$$

The travel distance δq of the DW t_p can be calculated by

$$\delta q = \int_0^{t_\mathrm{p}} \dot{q}\, dt\,. \tag{30}$$

With

$$
\begin{aligned}
f\left(M_{\mathrm{L}}, M_{\mathrm{U}}\right) =& \frac{\mp \frac{\pi}{2} \frac{\mu_{\mathrm{B}}\, j_{\mathrm{UL}}}{e} \frac{\theta_{\mathrm{L}}^{\mathrm{SH}}}{\gamma_{\mathrm{L}}}}{\frac{M_{\mathrm{L}}\, t_{\mathrm{L}}\, \alpha_{\mathrm{L}}}{\gamma_{\mathrm{L}}\, \Delta_{\mathrm{L}}} + \frac{M_{\mathrm{U}}\, t_{\mathrm{U}}\, \alpha_{\mathrm{U}}}{\gamma_{\mathrm{U}}\, \Delta_{\mathrm{U}}}} \\
g\left(M_{\mathrm{L}}, M_{\mathrm{U}}\right) =& \frac{\pm \frac{\pi}{2} \frac{\mu_{\mathrm{B}}\, j_{\mathrm{UL}}}{e} \frac{\theta_{\mathrm{U}}^{\mathrm{SH}}}{\gamma_{\mathrm{U}}}}{\frac{M_{\mathrm{L}}\, t_{\mathrm{L}}\, \alpha_{\mathrm{L}}}{\gamma_{\mathrm{L}}\, \Delta_{\mathrm{L}}} + \frac{M_{\mathrm{U}}\, t_{\mathrm{U}}\, \alpha_{\mathrm{U}}}{\gamma_{\mathrm{U}}\, \Delta_{\mathrm{U}}}} \\
h\left(M_{\mathrm{L}}, M_{\mathrm{U}}\right) =& \frac{\frac{M_{\mathrm{L}}\, t_{\mathrm{L}}}{\gamma_{\mathrm{L}}}}{\frac{M_{\mathrm{L}}\, t_{\mathrm{L}}\, \alpha_{\mathrm{L}}}{\gamma_{\mathrm{L}}\, \Delta_{\mathrm{L}}} + \frac{M_{\mathrm{U}}\, t_{\mathrm{U}}\, \alpha_{\mathrm{U}}}{\gamma_{\mathrm{U}}\, \Delta_{\mathrm{U}}}} \\
k\left(M_{\mathrm{L}}, M_{\mathrm{U}}\right) =& \frac{\frac{M_{\mathrm{U}}\, t_{\mathrm{U}}}{\gamma_{\mathrm{U}}}}{\frac{M_{\mathrm{L}}\, t_{\mathrm{L}}\, \alpha_{\mathrm{L}}}{\gamma_{\mathrm{L}}\, \Delta_{\mathrm{L}}} + \frac{M_{\mathrm{U}}\, t_{\mathrm{U}}\, \alpha_{\mathrm{U}}}{\gamma_{\mathrm{U}}\, \Delta_{\mathrm{U}}}}
\end{aligned}
\tag{31}
$$

we can write

$$
\begin{aligned}
\delta q = \int_0^{t_{\mathrm{p}}} & \left[f\left(M_{\mathrm{L}}, M_{\mathrm{U}}\right) \cos\phi_{\mathrm{L}} + g\left(M_{\mathrm{L}}, M_{\mathrm{U}}\right) \cos\phi_{\mathrm{U}} + \right. \\
& \left. h\left(M_{\mathrm{L}}, M_{\mathrm{U}}\right) \dot{\phi}_{\mathrm{L}} + k\left(M_{\mathrm{L}}, M_{\mathrm{U}}\right) \dot{\phi}_{\mathrm{U}}\right] dt\,.
\end{aligned}
\tag{32}
$$

If $m_i(t)$ was constant over time, the traveled distance can be calculated by

$$
\begin{aligned}
\delta q = & \left[f\left(M_{\mathrm{L}}, M_{\mathrm{U}}\right) \int_0^{t_{\mathrm{p}}} \cos\phi_{\mathrm{L}}\, dt + g\left(M_{\mathrm{L}}, M_{\mathrm{U}}\right) \int_0^{t_{\mathrm{p}}} \cos\phi_{\mathrm{U}}\, dt \right. \\
& \left. + h\left(M_{\mathrm{L}}, M_{\mathrm{U}}\right) \int_0^{t_{\mathrm{p}}} \dot{\phi}_{\mathrm{L}}\, dt + k\left(M_{\mathrm{L}}, M_{\mathrm{U}}\right) \int_0^{t_{\mathrm{p}}} \dot{\phi}_{\mathrm{U}}\, dt\right].
\end{aligned}
\tag{33}
$$

In contrast, we consider the case that $m_i(T)$ varies with time. We assume that ϕ_i and $\dot{\phi}_i$ vary very slowly during most of time during t_{p}. Indeed, due to the vary large exchange coupling in Co and Gd, the relaxation times of ϕ_i and $\dot{\phi}_i$ are so fast that this condition is fulfilled (c.f. [5]). In this case we can approximate δq by

$$
\begin{aligned}
\delta q \approx & \left[\int_0^{t_{\mathrm{p}}} f\left(M_{\mathrm{L}}, M_{\mathrm{U}}\right) dt\right]\left[\int_0^{t_{\mathrm{p}}} \cos\phi_{\mathrm{L}}\, dt\right] + \left[\int_0^{t_{\mathrm{p}}} g\left(M_{\mathrm{L}}, M_{\mathrm{U}}\right) dt\right]\left[\int_0^{t_{\mathrm{p}}} \cos\phi_{\mathrm{U}}\, dt\right] \\
& + \left[\int_0^{t_{\mathrm{p}}} h\left(M_{\mathrm{L}}, M_{\mathrm{U}}\right) dt\right]\left[\int_0^{t_{\mathrm{p}}} \dot{\phi}_{\mathrm{L}}\, dt\right] + \left[\int_0^{t_{\mathrm{p}}} k\left(M_{\mathrm{L}}, M_{\mathrm{U}}\right) dt\right]\left[\int_0^{t_{\mathrm{p}}} \dot{\phi}_{\mathrm{U}}\, dt\right]
\end{aligned}
\tag{34}
$$

In order to calculate the velocity v for this pulse, δq has to be divided by t_{p}. Thus,

$$
\begin{aligned}
v = & \bar{f}\left(M_{\mathrm{L}}, M_{\mathrm{U}}\right)\left[\int_0^{t_{\mathrm{p}}} \cos\phi_{\mathrm{L}}\, dt\right] + \bar{g}\left(M_{\mathrm{L}}, M_{\mathrm{U}}\right)\left[\int_0^{t_{\mathrm{p}}} \cos\phi_{\mathrm{U}}\, dt\right] \\
& + \bar{h}\left(M_{\mathrm{L}}, M_{\mathrm{U}}\right)\left[\int_0^{t_{\mathrm{p}}} \dot{\phi}_{\mathrm{L}}\, dt\right] + \bar{k}\left(M_{\mathrm{L}}, M_{\mathrm{U}}\right)\left[\int_0^{t_{\mathrm{p}}} \dot{\phi}_{\mathrm{U}}\, dt\right]
\end{aligned}
\tag{35}
$$

with $\bar{X}\left(M_{\mathrm{L}}, M_{\mathrm{U}}\right) = \frac{1}{t_{\mathrm{p}}} \int_0^{t_{\mathrm{p}}} X\left(M_{\mathrm{L}}, M_{\mathrm{U}}\right) dt$. When comparing Supplementary Equations (33) and (35), we can see that the DW displacement over time t_{p} almost equals in the case of time averaged moments and in case the moments change with time. Therefore, it is valid to calculate the DW velocity at an average temperature and compare these results with the measured DW velocity at the averaged temperature presented in Fig. 2 in the manuscript.

Supplementary Note 7 DW velocity dependence on A_{L} and A_{U} and j

We define $\mathrm{A}_i = \frac{m_i}{\gamma_i}$. We use $J_i^{\mathrm{pinning}} = \frac{\pi}{2} {m_i}^2\, K_i^{\mathrm{df}} \frac{\dot{q}}{|\dot{q}|}$ with $[J_i^{\mathrm{pinning}}] = \mathrm{J\ m^{-2}}$, $J^{\mathrm{SH}} = J_{\mathrm{U}}^{\mathrm{SH}} + J_{\mathrm{L}}^{\mathrm{SH}}$, $J^{\mathrm{DMI}} = J_{\mathrm{U}}^{\mathrm{DMI}} + J_{\mathrm{L}}^{\mathrm{DMI}}$ and $J^{\mathrm{pinning}} = J_{\mathrm{U}}^{\mathrm{pinning}} + J_{\mathrm{L}}^{\mathrm{pinning}}$. We will shorten the equations for ↑↓ and ↓↑ DWs by $\phi_{\substack{\mathrm{U}\\\mathrm{L}}}$ where the upper index corresponds to a ↑↓ and ↓↑ DW, respectively. The following approximations are made: $P = 0$, $\Delta_{\mathrm{U}} = \Delta_{\mathrm{L}} = \Delta$ and $\delta\phi \ll 1$. The latter is true for $J^{\mathrm{SH}} \ll J^{\mathrm{ex}}$ and $J^{\mathrm{DMI}} \ll J^{\mathrm{ex}}$. We assume $H_x = H_y = H_z = 0$.

With these approximations we can simplify Supplementary Equations (10), (11) and (12), as follows:

$$\dot{q} = \frac{1}{\zeta_{\mathrm{L}} + \zeta_{\mathrm{U}}} \left[J^{\mathrm{SH}} \cos \phi_{\substack{\mathrm{U}\\ \mathrm{L}}} - J^{\mathrm{pinning}} \right] \tag{36}$$

$$\dot{q} = \mp \frac{\alpha_{\mathrm{L}}}{\zeta_{\mathrm{L}}} \left[-J_{\mathrm{L}}^{\mathrm{DMI}} \sin \phi_{\substack{\mathrm{U}\\ \mathrm{L}}} + J^{\mathrm{ex}} \delta\phi \right] \tag{37}$$

$$\dot{q} = \mp \frac{\alpha_{\mathrm{U}}}{\zeta_{\mathrm{U}}} \left[J_{\mathrm{U}}^{\mathrm{DMI}} \sin \phi_{\substack{\mathrm{U}\\ \mathrm{L}}} + J^{\mathrm{ex}} \delta\phi \right] \tag{38}$$

These equations can be solved with respect to $\dot{q}$ without any $\delta\phi$ and $\phi_{\substack{\mathrm{U}\\ \mathrm{L}}}$ dependence. Thus, a closed form for $\dot{q}$ can be found.

Solving Supplementary Equation (37) for $\delta\phi$ and inserting this into Supplementary Equation (38) gives

$$\sin \phi_{\substack{\mathrm{U}\\ \mathrm{L}}} = \mp \frac{1}{J^{\mathrm{DMI}}} \left(\frac{\zeta_{\mathrm{U}}}{\alpha_{\mathrm{U}}} - \frac{\zeta_{\mathrm{L}}}{\alpha_{\mathrm{L}}} \right) \dot{q} \tag{39}$$

This can be inserted in Supplementary Equation (36) such that

$$\dot{q} = \frac{1}{\zeta_{\mathrm{L}} + \zeta_{\mathrm{U}}} \left\{ J^{\mathrm{SH}} \sqrt{1 - \left[\frac{1}{J^{\mathrm{DMI}}} \left(\frac{\zeta_{\mathrm{U}}}{\alpha_{\mathrm{U}}} - \frac{\zeta_{\mathrm{L}}}{\alpha_{\mathrm{L}}} \right) \dot{q} \right]^2} - J^{\mathrm{pinning}} \right\}. \tag{40}$$

Solving this equation in respect to $\dot{q}$ leads to

$$\begin{aligned} \dot{q} = & \frac{J^{\mathrm{SH}} \sqrt{(\zeta_{\mathrm{L}} + \zeta_{\mathrm{U}})^2 + \left(\frac{\zeta_{\mathrm{U}}}{\alpha_{\mathrm{U}}} - \frac{\zeta_{\mathrm{L}}}{\alpha_{\mathrm{L}}} \right)^2 \frac{J^{\mathrm{SH}2} - J^{\mathrm{pinning}2}}{J^{\mathrm{DMI}2}}}}{(\zeta_{\mathrm{L}} + \zeta_{\mathrm{U}})^2 + \left(\frac{\zeta_{\mathrm{U}}}{\alpha_{\mathrm{U}}} - \frac{\zeta_{\mathrm{L}}}{\alpha_{\mathrm{L}}} \right)^2 \frac{J^{\mathrm{SH}2}}{J^{\mathrm{DMI}2}}} \\ & - \frac{(\zeta_{\mathrm{L}} + \zeta_{\mathrm{U}}) J^{\mathrm{pinning}}}{(\zeta_{\mathrm{L}} + \zeta_{\mathrm{U}})^2 + \left(\frac{\zeta_{\mathrm{U}}}{\alpha_{\mathrm{U}}} - \frac{\zeta_{\mathrm{L}}}{\alpha_{\mathrm{L}}} \right)^2 \frac{J^{\mathrm{SH}2}}{J^{\mathrm{DMI}2}}} \end{aligned} \tag{41}$$

At the angular momentum compensation point where $\frac{m_{\mathrm{L}}}{\gamma_{\mathrm{L}}} = \frac{m_{\mathrm{U}}}{\gamma_{\mathrm{U}}}$, the DW velocity is given

$$\dot{q} = \frac{J^{\mathrm{SH}} - J^{\mathrm{pinning}}}{(\zeta_{\mathrm{L}} + \zeta_{\mathrm{U}})}. \tag{42}$$

Supplementary Equation (41) can be approximated in the limit of no pinning which leads to a simple form for the velocity at any $\mathrm{A_U}$ and $\mathrm{A_L}$ that is given by:

$$\dot{q} = \left\{ \left[\frac{\mathrm{A_U} - \mathrm{A_L}}{\frac{\pi}{2} (D_{\mathrm{L}} + D_{\mathrm{U}})} \right]^2 + \left[\frac{\mathrm{A_U}\, \alpha_{\mathrm{U}} + \mathrm{A_L} \alpha_{\mathrm{L}}}{\Delta \left(J_{\mathrm{U}}^{\mathrm{SH}} + J_{\mathrm{L}}^{\mathrm{SH}} \right)} \right]^2 \right\}^{-\frac{1}{2}} \operatorname{sgn}(j). \tag{43}$$

Using this equation we can show that there is a maximum velocity at a certain ratio $R^* = \mathrm{A_L}/\mathrm{A_U}$ for high current densities as was experimentally observed. For the case of Co and Gd, $\frac{dm_{\mathrm{L}}}{dT} \ll \frac{dm_{\mathrm{U}}}{dT}$ so that we assume that only $\mathrm{A_U}$ is changing with temperature. To find the maximum we set:

$$\begin{aligned} \frac{d\dot{q}}{d\mathrm{A_U}} = & - \left\{ \frac{\mathrm{A_U^*} - \mathrm{A_L}}{\left[\frac{\pi}{2} (D_{\mathrm{L}} + D_{\mathrm{U}}) \right]^2} + \alpha_{\mathrm{U}} \frac{\mathrm{A_U^*}\, \alpha_{\mathrm{U}} + \mathrm{A_L} \alpha_{\mathrm{L}}}{\left[\Delta \left(J_{\mathrm{U}}^{\mathrm{SH}} + J_{\mathrm{L}}^{\mathrm{SH}} \right) \right]^2} \right\} \\ & \times \left\{ \left[\frac{\mathrm{A_U^*} - \mathrm{A_L}}{\frac{\pi}{2} (D_{\mathrm{L}} + D_{\mathrm{U}})} \right]^2 + \left[\frac{\mathrm{A_U^*}\, \alpha_{\mathrm{U}} + \mathrm{A_L} \alpha_{\mathrm{L}}}{\Delta \left(J_{\mathrm{U}}^{\mathrm{SH}} + J_{\mathrm{L}}^{\mathrm{SH}} \right)} \right]^2 \right\}^{-\frac{3}{2}} = 0 \end{aligned} \tag{44}$$

which is fulfilled if,

$$\frac{\mathrm{A_U^*} - \mathrm{A_L}}{J^{\mathrm{DMI}2}} + \alpha_{\mathrm{U}} \frac{\mathrm{A_U^*}\, \alpha_{\mathrm{U}} + \mathrm{A_L} \alpha_{\mathrm{L}}}{J^{\mathrm{SH}2}} = 0. \tag{45}$$

Thus, at the ratio

$$R^* = \frac{{J^{\rm SH}}^2 + \alpha_{\rm U}^2\, {J^{\rm DMI}}^2}{{J^{\rm SH}}^2 - \alpha_{\rm U}\, \alpha_{\rm L}\, {J^{\rm DMI}}^2} \tag{46}$$

the DW velocity takes a maximum value.

On the one hand, this result shows that as the current density increases the maximum in the DW velocity shifts toward the angular momentum compensation point. On the other hand, if the current density is small when $\left(J_{\rm L}^{\rm SH} + J_{\rm U}^{\rm SH}\right)^2 < \alpha^2 {J_{\rm L}^{\rm DMI}}^2$ there is no local maximum and the DW velocity monotonically decreases with decreasing temperature. In this situation the maximum will be at $R^* \to \infty$. These theoretical results are consistent with what we observe in our experiments.

We note that Supplementary Equation (46) is not valid for $j_{\rm UL} \to \infty$ as $\delta\phi \ll 1$ is not fulfilled in this case. However, under the conditions that $\theta_{\rm U}^{\rm SH} \approx 0$ and $M_{\rm L}(T)$ is constant, which are to some degree valid for our Co/Gd bi-layer, we find Supplementary Equation (46) to be the exact solution for the optimal ratio. In this case it is valid to claim that the DW velocity maximum converges exactly to the angular momentum compensation temperature. Additionally, we tested the influence of pinning (cf. model explained in Supplementary Note 4) by numerical simulations. With the parameters which we used, there is only a small effect of pinning on R^*.

Supplementary Note 8 Solutions for special cases

The following section investigates the special cases of 1) total angular momentum compensation where $\frac{M_{\rm L}\, t_{\rm L}}{\gamma_{\rm L}} = \frac{M_{\rm U}\, t_{\rm U}}{\gamma_{\rm U}}$ and 2) the domination of one layer where $\frac{M_{\rm L}\, t_{\rm L}}{\gamma_{\rm L}} \gg \frac{M_{\rm U}\, t_{\rm U}}{\gamma_{\rm U}}$ or $\frac{M_{\rm L}\, t_{\rm L}}{\gamma_{\rm L}} \ll \frac{M_{\rm U}\, t_{\rm U}}{\gamma_{\rm U}}$. This section refers to Supplementary Equations (10), (11) and (12). The focus is on the temperature and current density dependence which we find in the experiments. Pinning is not considered in the following discussion. Note that in case 1) $\zeta_L = \zeta_{\rm U}$ and in case 2) $\zeta_{\rm L} \gg \zeta_U$ and $\zeta_{\rm L} \ll \zeta_U$.

Supplementary Equation (10) can be rewritten with the above mentioned assumptions to

$$\dot{q} = \frac{1}{\zeta_{\rm L} + \zeta_{\rm U}} \left[\mp J_{\rm L}^{\rm SH} \cos\phi_{\rm L} \pm J_{\rm U}^{\rm SH} \cos\phi_{\rm U}\right]. \tag{47}$$

Supplementary Equation (11) can be rewritten to

$$\dot{q} = \mp \frac{\alpha_{\rm L}}{\zeta_{\rm L}} \left[\pm J_{\rm L}^{\rm DMI} \sin\phi_{\rm L} - J^{\rm ex} \sin(\phi_{\rm L} - \phi_{\rm U})\right] \tag{48}$$

Supplementary Equation (12) can be rewritten to

$$\dot{q} = \pm \frac{\alpha_{\rm U}}{\zeta_{\rm U}} \left[J^{\rm ex} \sin(\phi_{\rm L} - \phi_{\rm U})\right]. \tag{49}$$

Equating Supplementary Equations (48) and (49) will lead to[1]

$$\phi_{\rm L} = \begin{cases} \pi \text{ or } 0 & \text{for an } \uparrow\downarrow \text{ or } \downarrow\uparrow \text{ DW, respectively, in case 1)} \\ \phi_{\rm U} + \pi & \text{in case 2).} \end{cases} \tag{50}$$

In order to solve these equations (excluding ϕ_i dependence) a few approximations will be needed for the values of $J^{\rm ex}$, $J_i^{\rm SH}$ and $J_{\rm L}^{\rm DMI}$. $J^{\rm ex}$ is in the range $1 - 2$ mJ m^{-2}. $j_{\rm UL}$ is 3×10^8 A cm^{-2} at maximum which gives an upper limit for $J_i^{\rm SH}$ of about 0.3 mJ m^{-2} for $\theta_i^{\rm SH} < 0.2$. With $D_{\rm L}$ in the order of $0.1 - 3$ pJ and Δ between $2 - 10$ nm, $J_{\rm L}^{\rm DMI}$ is in the range $0.02 - 2.3$ mJ m^{-2}. Note that in our samples $D_{\rm L}$ is quite small so that $J_{\rm L}^{\rm DMI}$ is about 0.14 mJ m^{-2}.

8.1 Case 1: Total angular momentum compensation

In this case $\zeta_{\rm L} = \zeta_{\rm U} = \zeta$. Solving Supplementary Equations (48) and (49) together, one can find $\sin\phi_{\rm L} = 0$ which gives $\phi_{\rm L} = 0$ or π. Using Supplementary Equation (50) will reduce Supplementary Equation (47) to

$$\dot{q} = \frac{1}{2\,\zeta} \left(J_{\rm L}^{\rm SH} \pm J_{\rm U}^{\rm SH} \cos\phi_{\rm U}\right) \tag{51}$$

[1] In case of 2) and strong DMI ($J^{\rm ex} \sim J_{\rm L}^{\rm DMI}$), $\phi_{\rm L} = \pi$ or 0 for an $\uparrow\downarrow$ or $\downarrow\uparrow$ DW applies, respectively.

as well as Supplementary Equation (49) to

$$\dot{q} = \pm\frac{\alpha}{\zeta} J^{\text{ex}} \sin\phi_{\text{U}}. \tag{52}$$

By assuming a large exchange coupling between the two layers $\left(J^{\text{ex}} \gg \frac{1}{2\,\alpha} J_i^{\text{SH}}\right)$, this leads to the solution

$$\dot{q} = \frac{1}{2\,\zeta}\left(J_{\text{L}}^{\text{SH}} + J_{\text{U}}^{\text{SH}}\right). \tag{53}$$

This result can be interpreted by looking at Supplementary Equation (9) as follows. Due to $\sin\phi_{\text{L}} = 0$ the DMI term in Supplementary Equation (9) goes to zero but $\sin\phi_{\text{U}}$ will be just as high that Supplementary Equations (51) and (52) are fulfilled. Thus, at the total angular momentum compensation point, the DW is no longer driven by the torque arising from the DMI but instead by the exchange coupling torque.

Comparing these results to field-driven DW motion [14], one can find that at T_{A} the DW can be driven at the same speed by an external field

$$H_z = \frac{\pi}{2}\frac{H_{\text{Total,L}}^{\text{SH}}}{\left(1 - \frac{\gamma_{\text{U}}}{\gamma_{\text{L}}}\right)} \tag{54}$$

with $H_{\text{Total,L}}^{\text{SH}} = \frac{\mu_{\text{B}}\, j_{\text{UL}}}{m_{\text{L}}\, e}\left(\frac{\theta_{\text{L}}^{\text{SH}}}{\gamma_{\text{L}}} + \frac{\theta_{\text{U}}^{\text{SH}}}{\gamma_{\text{U}}}\right)$. This indicates the efficiency differences of field-driven DW motion and CIDWM at T_{A}. Particularly, if γ_{U} is close to γ_{L}, a much larger H_z is required to achieve the same DW velocity as for CIDWM. Especially in case of SAF structures, for which $T_{\text{A}} = T_{\text{M}}$, the DW cannot be moved by any H_z but instead can by efficiently moved by a spin Hall current.

We would like to compare these results also with [15] which has been published during the review process. Supplementary Equations (10), (11) and (12) can be rewritten for strong coupling ($\phi_{\text{U}} \approx \phi_{\text{L}}+\pi$) using $A_{\text{net}} = A_{\text{L}} - A_{\text{U}}$, $\dot{\phi}_{\substack{\text{L}\\\text{U}}} = \varrho\,\dot{\phi}_{\substack{\text{U}\\\text{L}}}$ (where ϱ might be either $+1$ or -1), $\alpha_{\text{eff}} = \frac{\alpha_{\text{L}}\, A_{\text{L}} + \alpha_{\text{U}}\, A_{\text{U}}}{A_{\text{net}}}$ and $A_{\text{net}}^{\varrho} = A_{\text{U}} - \varrho\, A_{\text{L}}$ in the following way

$$\frac{\alpha_{\text{eff}}\, A_{\text{net}}}{\Delta}\,\dot{q} = J^{\text{SH}}\cos\phi_{\substack{\text{U}\\\text{L}}} + A_{\text{net}}^{\varrho}\dot{\phi}_{\substack{\text{U}\\\text{L}}} \tag{55}$$

$$\mp\frac{A_{\text{L}}}{\Delta}\,\dot{q} = -J_{\text{L}}^{\text{DMI}}\sin\phi_{\substack{\text{U}\\\text{L}}} + J^{\text{ex}}\,\delta\phi \pm^{\varrho} \alpha_{\text{L}}\, A_{\text{L}}\,\dot{\phi}_{\substack{\text{U}\\\text{L}}} \tag{56}$$

$$\mp\frac{A_{\text{U}}}{\Delta}\,\dot{q} = J_{\text{U}}^{\text{DMI}}\sin\phi_{\substack{\text{U}\\\text{L}}} + J^{\text{ex}}\,\delta\phi \pm_{\varrho} \alpha_{\text{U}}\, A_{\text{U}}\,\dot{\phi}_{\substack{\text{U}\\\text{L}}} \tag{57}$$

where $\pm^{\varrho}$ means that ϱ has to be multiplied with the upper sign and $\pm_{\varrho}$ means that ϱ has to be multiplied with the lower sign. By solving Supplementary Equation (56) or (57) in respect to $J^{\text{ex}}\,\delta\phi$ and inserting into the other gives

$$\dot{\phi}_{\substack{\text{U}\\\text{L}}} = \mp^{\varrho}\frac{1}{\alpha_{\text{L}}\, A_{\text{L}} + \varrho\,\alpha_{\text{U}} A_{\text{U}}}\left(\pm\frac{A_{\text{L}} - A_{\text{U}}}{\Delta}\dot{q} - J^{\text{DMI}}\sin\phi_{\substack{\text{U}\\\text{L}}}\right). \tag{58}$$

This can be inserted into Supplementary Equation (55) which results with $\varrho = +1$ in

$$\frac{A_{\text{net}}\left({\alpha_{\text{eff}}}^2 + 1\right)}{\Delta}\,\dot{q} = \alpha_{\text{eff}}\, J^{\text{SH}}\cos\phi_{\substack{\text{U}\\\text{L}}} \pm J^{\text{DMI}}\sin\phi_{\substack{\text{U}\\\text{L}}}. \tag{59}$$

This is the same result as in [15] if equations (1) and (2) in the SI of this paper are solved by inserting $\dot{\phi}$ into each other taking the different definition of ϕ into account and just looking at an ↓↑ DW which results in

$$\frac{A_{\text{net}}\left({\alpha_{\text{eff}}}^2 + 1\right)}{\Delta}\,\dot{q} = \alpha_{\text{eff}}\, J^{\text{SH}}\sin\phi - J^{\text{DMI}}\cos\phi. \tag{60}$$

8.2 Case 2: One dominant layer

Lower layer dominant

First, the situation of a dominant lower layer ($\frac{\zeta_{\text{U}}}{\zeta_{\text{L}}} \approx 0$) is considered because it directly results in equations which are equivalent to the well known single layer case. By solving Supplementary Equations (48) and (49) and using

this approximation, $\sin(\phi_{\mathrm{L}} - \phi_{\mathrm{U}}) \approx 0$ which is given if $\phi_{\mathrm{U}} = \phi_{\mathrm{L}} - \pi$. Inserting ϕ_{U} into Supplementary Equations (47) and (48), one arrives at the solution

$$\dot{q} = \frac{1}{\zeta_{\mathrm{L}}} \left(\alpha\, J_{\mathrm{L}}^{\mathrm{DMI}}\right) \frac{\left(J_{\mathrm{L}}^{\mathrm{SH}} + J_{\mathrm{U}}^{\mathrm{SH}}\right)}{\sqrt{\left(\alpha\, J_{\mathrm{L}}^{\mathrm{DMI}}\right)^2 + \left(J_{\mathrm{L}}^{\mathrm{SH}} + J_{\mathrm{U}}^{\mathrm{SH}}\right)^2}} \tag{61}$$

This equation can be rewritten and is equivalent to a single (lower) layer with an additional driving term by the spin Hall current in the upper magnetic layer know to be of the form

$$\dot{q} = \frac{\pi}{2} \frac{\Delta\, \gamma_{\mathrm{L}}}{\alpha} \frac{H_{\mathrm{L}}^{\mathrm{DMI}} \left(H_{\mathrm{L}}^{\mathrm{SH}} + H_{\mathrm{U}}^{\mathrm{SH}} \frac{M_{\mathrm{U}}\, t_{\mathrm{U}}}{M_{\mathrm{L}}\, t_{\mathrm{L}}}\right)}{\sqrt{{H_{\mathrm{L}}^{\mathrm{DMI}}}^2 + \frac{1}{\alpha^2} \left(H_{\mathrm{L}}^{\mathrm{SH}} + H_{\mathrm{U}}^{\mathrm{SH}} \frac{M_{\mathrm{U}}\, t_{\mathrm{U}}}{M_{\mathrm{L}}\, t_{\mathrm{L}}}\right)^2}}. \tag{62}$$

Be aware that the upper layers' contribution by the spin Hall current is not negligible as the ratio $\frac{M_{\mathrm{U}}\, t_{\mathrm{U}}}{M_{\mathrm{L}}\, t_{\mathrm{L}}}$ might suggest. A more straight forward expression might be

$$\dot{q} = \frac{\pi}{2} \frac{\Delta\, \gamma_{\mathrm{L}}}{\alpha} \frac{H_{\mathrm{L}}^{\mathrm{DMI}} H_{\mathrm{Total,L}}^{\mathrm{SH}}}{\sqrt{{H_{\mathrm{L}}^{\mathrm{DMI}}}^2 + \frac{1}{\alpha^2} {H_{\mathrm{Total,L}}^{\mathrm{SH}}}^2}}. \tag{63}$$

If the DMI was large compared to the applied current density $\alpha\, J_{\mathrm{L}}^{\mathrm{DMI}} \gg (J_{\mathrm{L}}^{\mathrm{SH}} + J_{\mathrm{U}}^{\mathrm{SH}})$, the velocity would be proportional to the current density:

$$\dot{q} = \frac{1}{\zeta_{\mathrm{L}}} \left(J_{\mathrm{L}}^{\mathrm{SH}} + J_{\mathrm{U}}^{\mathrm{SH}}\right). \tag{64}$$

Upper layer dominant

Finally, the situation is considered in which the upper layer is dominant. If the exchange coupling is large enough, meaning $J^{\mathrm{ex}} \gg J_{\mathrm{L}}^{\mathrm{DMI}}$, $\phi_{\mathrm{U}} = \phi_{\mathrm{L}} + \pi$ and the velocity will be given by

$$\dot{q} = \frac{1}{\zeta_{\mathrm{U}}} \left(\alpha\, J_{\mathrm{L}}^{\mathrm{DMI}}\right) \frac{\left(J_{\mathrm{L}}^{\mathrm{SH}} + J_{\mathrm{U}}^{\mathrm{SH}}\right)}{\sqrt{\left(\alpha\, J_{\mathrm{L}}^{\mathrm{DMI}}\right)^2 + \left(J_{\mathrm{L}}^{\mathrm{SH}} + J_{\mathrm{U}}^{\mathrm{SH}}\right)^2}} \tag{65}$$

or equivalently described as

$$\dot{q} = \frac{\pi}{2} \frac{\Delta\, \gamma_{\mathrm{U}}}{\alpha} \frac{H_{\mathrm{L}}^{\mathrm{DMI}} H_{\mathrm{Total,U}}^{\mathrm{SH}}}{\sqrt{{H_{\mathrm{L}}^{\mathrm{DMI}}}^2 + \frac{1}{\alpha^2} {H_{\mathrm{Total,U}}^{\mathrm{SH}}}^2}} \tag{66}$$

with $H_{\mathrm{Total,U}}^{\mathrm{SH}} = \frac{\mu_{\mathrm{B}}\, j_{\mathrm{UL}}}{M_{\mathrm{U}}\, t_{\mathrm{U}}\, e} \left(\frac{\theta_{\mathrm{L}}^{\mathrm{SH}}}{\gamma_{\mathrm{L}}} + \frac{\theta_{\mathrm{U}}^{\mathrm{SH}}}{\gamma_{\mathrm{U}}}\right)$. In this situation the upper layer is very much coupled to the lower layer but the DMI in the lower layer is comparably weak. The upper layer is therefore dependent on the DMI of the lower layer in order to achieve an efficient motion which results in a saturation of the velocity at higher current densities.

If the DMI was very large $J^{\mathrm{ex}} \sim J_{\mathrm{L}}^{\mathrm{DMI}}$ or the current density low such that $\alpha\, J_{\mathrm{L}}^{\mathrm{DMI}} \gg (J_{\mathrm{L}}^{\mathrm{SH}} + J_{\mathrm{U}}^{\mathrm{SH}})$, then ϕ_{U} would be 0 or π for an $\uparrow\downarrow$ or $\downarrow\uparrow$ DW, respectively and the final velocity would be given by

$$\dot{q} = \frac{1}{\zeta_{\mathrm{U}}} \left(J_{\mathrm{L}}^{\mathrm{SH}} + J_{\mathrm{U}}^{\mathrm{SH}}\right). \tag{67}$$

This result is equivalent to a single (upper) layer which experiences a comparably large DMI field $\left(H_{\mathrm{L}}^{\mathrm{DMI}} = \frac{D_{\mathrm{L}}}{\Delta_{\mathrm{L}}\, M_{\mathrm{L}}\, t_{\mathrm{L}}}\right)$ from the lower layer.

8.3 Scaling with current density

Interestingly, all equations show that the final velocity is dependent on the sum $J_{\mathrm{L}}^{\mathrm{SH}} + J_{\mathrm{U}}^{\mathrm{SH}}$. The velocity is determined by the total spin current thus independent of the ratio of spin currents on Co and Gd layers. There is just a scaling with γ_i as $J_{\mathrm{L}}^{\mathrm{SH}} + J_{\mathrm{U}}^{\mathrm{SH}} \propto \left(\frac{\theta_{\mathrm{L}}^{\mathrm{SH}}}{\gamma_{\mathrm{L}}} + \frac{\theta_{\mathrm{U}}^{\mathrm{SH}}}{\gamma_{\mathrm{U}}}\right)$.

In our samples we assume a low DMI so that $\alpha\, J_{\mathrm{L}}^{\mathrm{DMI}} \sim (J_{\mathrm{L}}^{\mathrm{SH}} + J_{\mathrm{U}}^{\mathrm{SH}})$ at high current densities and $J^{\mathrm{ex}} \gg J_{\mathrm{L}}^{\mathrm{DMI}}$. Whereas at the total angular momentum compensation point (Supplementary Equation (53)) the velocity scales linearly with the applied current, the velocity only scales like $v \propto \frac{j_{\mathrm{UL}}}{\sqrt{c^2 + j_{\mathrm{UL}}{}^2}}$ with c being constant if one layer is more dominant (Supplementary Equations (61) and (65)). This means that at high current densities ($c \ll j_{\mathrm{UL}}$) the motion at the angular momentum compensation temperature will be far more efficient. For low current densities where $\alpha\, J_{\mathrm{L}}^{\mathrm{DMI}} \gg (J_{\mathrm{L}}^{\mathrm{SH}} + J_{\mathrm{U}}^{\mathrm{SH}})$ the velocity will scale linearly with the applied current density at all temperatures.

If the upper layer is dominant, the scaling with current density also depends on the ratio of J^{ex} to $J_{\mathrm{L}}^{\mathrm{DMI}}$. This is because if the DMI energy in the lower layer is comparably small, the upper layer is restricted to that. But if the DMI energy is in the same order of the exchange energy, the velocity is no longer restricted by the DMI energy.

At the total angular momentum compensation temperature, the DMI is no longer the driving force but the exchange coupling torque instead. The exchange coupling torque is much more efficient than the DMI torque which we observed experimentally by temperature dependent CIDWM measurements. This effect cannot be seen in single magnetic layers (cf. section 8.2) in which a constant decline of the velocity occurs due to increase of $M(T)$ and increasing pinning while lowering the temperature.

In conculsion, the presented results clearly show that the total angular momentum is the relevant quantity in a bi-layer systems like Co/Gd and theoretically confirm the efficiency increase at T_{A}. Considering the temperature dependence of the magnetization of $M_{\mathrm{U}}(T)$ and $M_{\mathrm{L}}(T)$ discussed in section 1.2, one can find the extreme case 2) if $T < T_{\mathrm{A}}$ where $\zeta_{\mathrm{L}} \ll \zeta_{\mathrm{U}}$ as well as if $T > T_{\mathrm{A}}$ where $\zeta_{\mathrm{L}} \gg \zeta_{\mathrm{U}}$ and extreme case 1) if $T = T_{\mathrm{A}}$. Due to the difference in the scaling with current density in these regimes, the temperature dependence of the velocity will also depend on the applied current density. If the current density is low, the velocity will scale linearly with the applied current density in all regimes but also with $\frac{1}{\zeta_{\mathrm{L}} + \zeta_{\mathrm{U}}}$. Thus, if j_{UL} is constant but (especially) $M_{\mathrm{U}}(T)$ increases with decreasing temperature, the velocity will drop continuously. More interesting is the case where the current density is high such that $\alpha\, J_{\mathrm{L}}^{\mathrm{DMI}} \sim (J_{\mathrm{L}}^{\mathrm{SH}} + J_{\mathrm{U}}^{\mathrm{SH}})$. In this case the velocity will linearly increase with the current density at T_{A} but not away from T_{A}.

Supplementary Figures

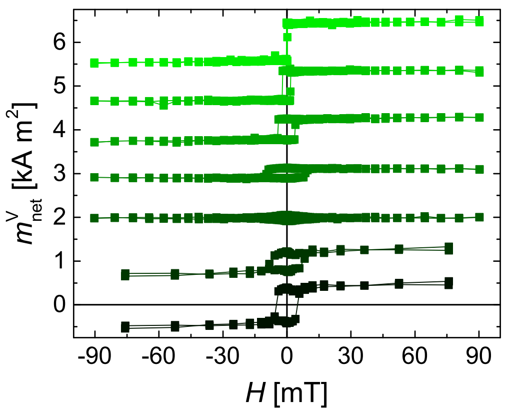

Supplementary Figure 1: **Out-of-plane hysteresis loops of a Co/Gd bi-layer.** Measured at various temperatures near T_{M}. Temperature is varied in 10 K steps from 190 K to 250 K (color coded from black to light green). Graph shows the net moment of the whole sample volume, denoted as $m_{\mathrm{net}}^{\mathrm{V}}$. The loops are displaced vertically for clarity.

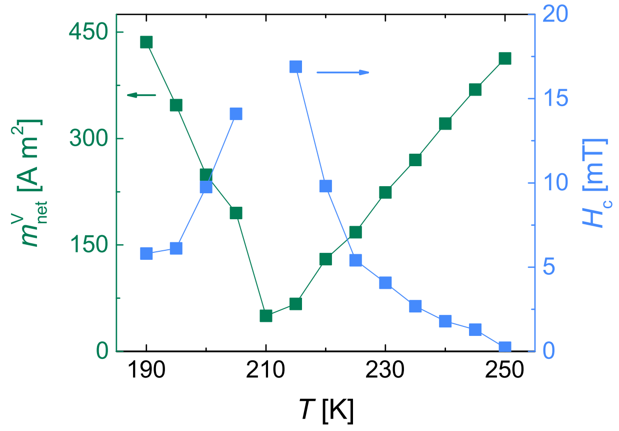

Supplementary Figure 2: **Magnetic properties of ferrimagnetic bilayer.** Magnetic net moment of whole sample volume $m_{\mathrm{net}}^{\mathrm{V}}$ and coercive field H_c as a function of temperature T from SQUID magnetometry.

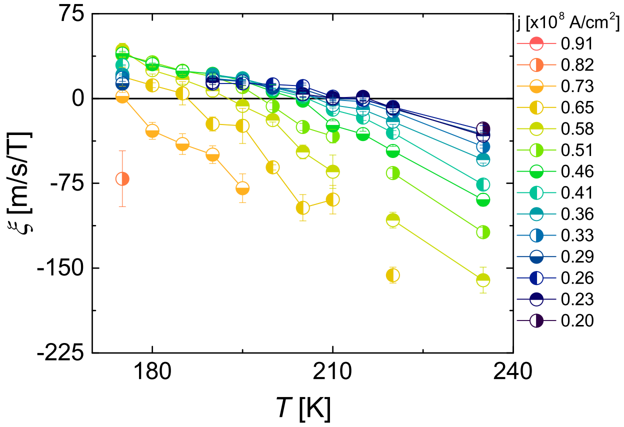

Supplementary Figure 3: **Slope of H_x dependence vs. cryostat temperature for pulse length of 20 ns.** Error bars reflect fitting error of H_x-dependent velocity to linear fit (c.f. Fig. 2a in manuscript).

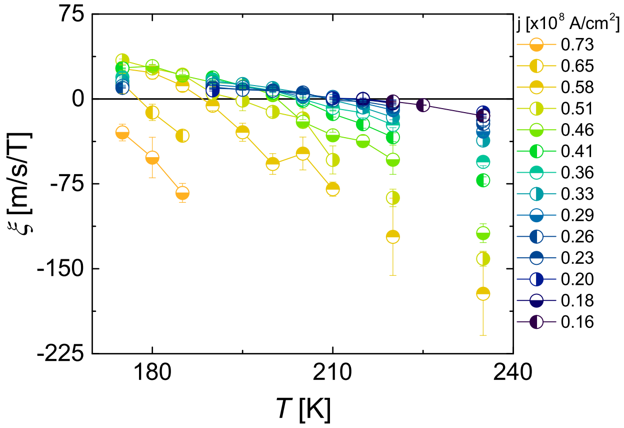

Supplementary Figure 4: **Slope of H_x dependence vs. cryostat temperature for pulse length of 40 ns.** Error bars reflect fitting error of H_x-dependent velocity to linear fit (c.f. Fig. 2a in manuscript).

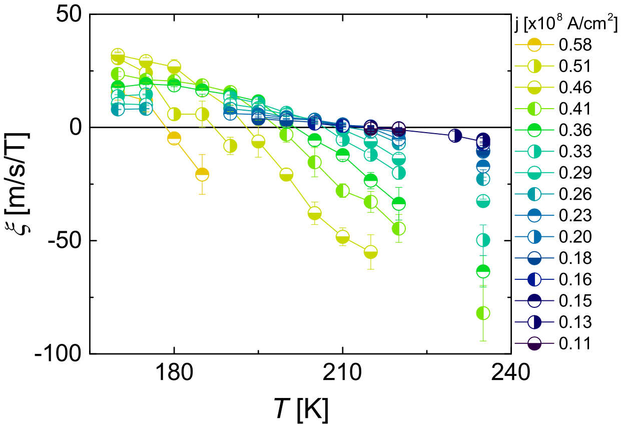

Supplementary Figure 5: Slope of H_x dependence vs. cryostat temperature for pulse length of 100 ns. Error bars reflect fitting error of H_x-dependent velocity to linear fit (c.f. Fig. 2a in manuscript).

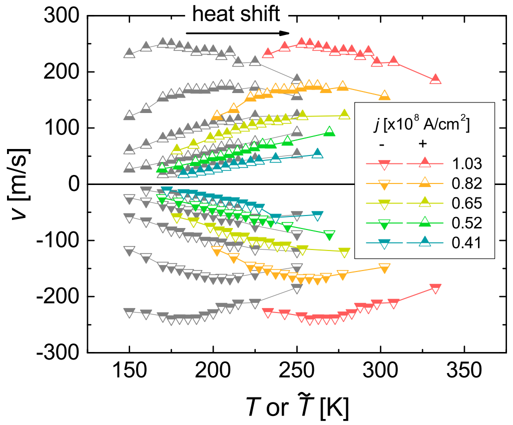

Supplementary Figure 6: Heat shifted data. Experimentally measured DW velocity v versus cryostat temperature T (grey) and heat shifted data $\tilde{T}$ (colored). Due to heating during the pulse, T is elevated by $\delta T_{\mathrm{cur}}(j) =$ 83 K, 52 K, 28 K, 19 K, 12 K for j from 1.03 to 0.41 × 10^8 A cm^{-2}.

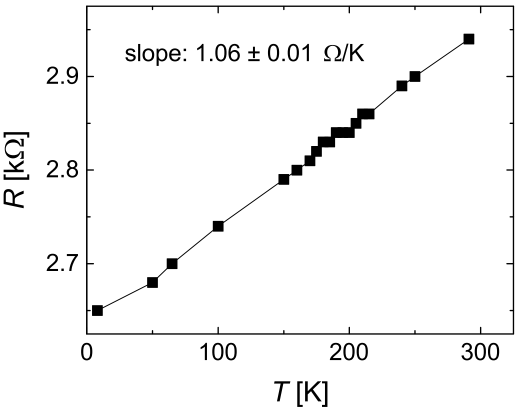

Supplementary Figure 7: **Resistance of CIDWM device as a function of temperature.**

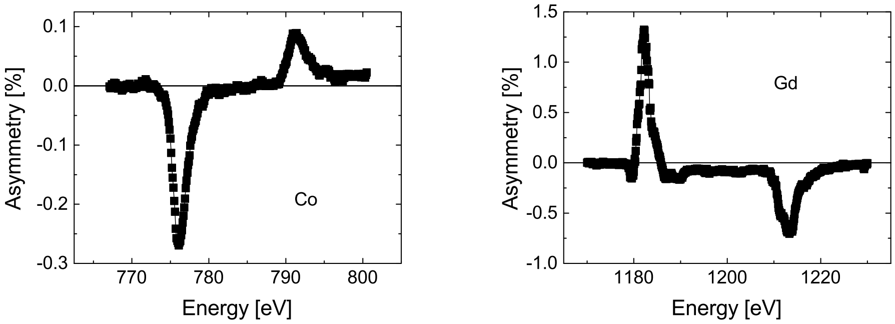

Supplementary Figure 8: **XMCD spectra of Co.** Measured at L2 and L3 edges (left), and Gd at M4 and M5 edges (right) at T = 222.5 K.

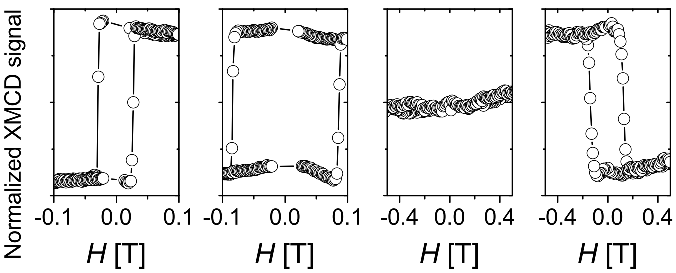

Supplementary Figure 9: **Temperature dependent magnetic hysteresis loops of Gd.** Left to right: T = 190 K, 205 K, 207.5 K, 210 K

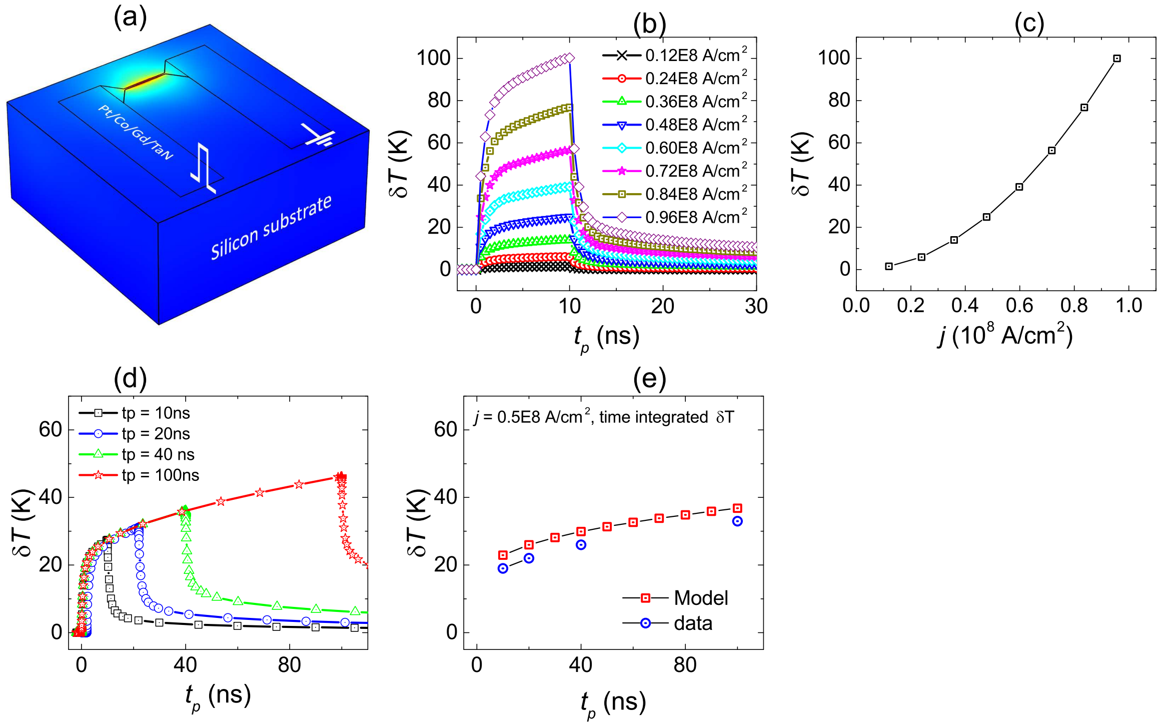

Supplementary Figure 10: **Simulated temperature dependence of device during current pulse.** (a) Three-dimensional temperature profile of the racetrack on a Silicon substrate [here red (blue) is hot(cold)]. A rectangular voltage pulse V_0 with a variable pulse duration t_p is applied to the left leg of the racetrack while the right-leg is kept at the ground potential. Most of the power is dissipated in the narrower-section which shows an elevated temperature. (b) The temperature $\delta T = T - T_{ref}$, where T_{ref} is the surrounding temperature rise as a function of time (in nanoseconds) different current densities j with a pulse duration t_p of 10 ns (c) The maximum temperature rise as a function of the current density for the data in (b), (d) Pulse duration dependence of the temperature rise and (e) the time-averaged increase for the data in (d).

Supplementary Tables

Material	σ [10^6 S m^{-1}]	C_p [J kg^{-1} K^{-1}]	ρ [kg m^{-3}]	κ [W mK^{-1}]
Racetrack[1]	2.3	138	8400	11
SiO_2	10^{-12}	423	3300	1.5
Si	10^{-3}	420	2300	200

Supplementary Table 1: Material parameters used for the finite-element simulation at $T_{\mathrm{ref}} = 210$ K. The material parameters for Si, TaN and SiO_2 are taken from [16, 17, 18, 19, 13] as well as the built-in COMSOL material library.

Supplementary References

[1] Charles Kittel. *Introduction to Solid State Physics*, volume 8. John Wiley & Son, Inc, 2005.

[2] C. M. Schneider, P. Bressler, P. Schuster, J. Kirschner, J. J. Demiguel, and R. Miranda. Curie-temperature of ultrathin films of fcc cobalt epitaxially grown on atomically flat cu(100) surfaces. *Physical Review Letters*, 64(9):1059–1062, 1990.

[3] M. Farle, W. A. Lewis, and K. Baberschke. Detailed analysis of the insitu magnetooptic kerr signal of gadolinium films near the curie-temperature. *Applied Physics Letters*, 62(21):2728–2730, 1993.

[4] T. H. Pham, J. Vogel, J. Sampaio, M. Vanatka, J. C. Rojas-Sanchez, M. Bonfim, D. S. Chaves, F. Choueikani, P. Ohresser, E. Otero, A. Thiaville, and S. Pizzini. Very large domain wall velocities in pt/co/gdox and pt/co/gd trilayers with dzyaloshinskii-moriya interaction. *Epl*, 113(6):67001, 2016.

[5] S. H. Yang, K. S. Ryu, and S. Parkin. Domain-wall velocities of up to 750 m s(-1) driven by exchange-coupling torque in synthetic antiferromagnets. *Nature Nanotechnology*, 10(3):221–226, 2015.

[6] S. H. Yang and S. Parkin. Novel domain wall dynamics in synthetic antiferromagnets. *Journal of Physics-Condensed Matter*, 29(30):303001, 2017.

[7] F. J. A. Denbroeder, W. Hoving, and P. J. H. Bloemen. Magnetic-anisotropy of multilayers. *Journal of Magnetism and Magnetic Materials*, 93:562–570, 1991.

[8] R. Hasegawa. Static bubble-domain properties of amorphous gd-co films. *Journal of Applied Physics*, 45(7):3109–3112, 1974.

[9] J. Seib and M. Fahnle. Calculation of the gilbert damping matrix at low scattering rates in gd. *Physical Review B*, 82(6):064401, 2010.

[10] K. S. Ryu, L. Thomas, S. H. Yang, and S. Parkin. Chiral spin torque at magnetic domain walls. *Nature Nanotechnology*, 8(7):527–533, 2013.

[11] J. Finley and L. Q. Liu. Spin-orbit-torque efficiency in compensated ferrimagnetic cobalt-terbium alloys. *Physical Review Applied*, 6(5):054001, 2016.

[12] G. Consolo and E. Martinez. The effect of dry friction on domain wall dynamics: A micromagnetic study. *Journal of Applied Physics*, 111(7):07D312, 2012.

[13] David G. Cahill, Wayne K. Ford, Kenneth E. Goodson, Gerald D. Mahan, Arun Majumdar, Humphrey J. Maris, Roberto Merlin, and Simon R. Phillpot. Nanoscale thermal transport. *Journal of Applied Physics*, 93(2):793–818, 2003.

[14] K. J. Kim, S. K. Kim, Y. Hirata, S. H. Oh, T. Tono, D. H. Kim, T. Okuno, W. S. Ham, S. Kim, G. Go, Y. Tserkovnyak, A. Tsukamoto, T. Moriyama, K. J. Lee, and T. Ono. Fast domain wall motion in the vicinity of the angular momentum compensation temperature of ferrimagnets. *Nature Materials*, 16(12):1187–1192, 2017.

[15] Saima A. Siddiqui, Jiahao Han, Joseph T. Finley, Caroline A. Ross, and Luqiao Liu. Current-induced domain wall motion in a compensated ferrimagnet. *Phys. Rev. Lett.*, 121:057701, Jul 2018.

[16] C. J. Glassbrenner and Glen A. Slack. Thermal conductivity of silicon and germanium from 3 k to the melting point. *Phys. Rev.*, 134(4A):A1058–A1069, May 1964.

[17] Glen A. Slack. Thermal Conductivity of Pure and Impure Silicon, Silicon Carbide, and Diamond. *Journal of Applied Physics*, 35(12):3460–3466, December 1964.

[18] A. S. Okhotin, L. I. Zhmakin, and A. P. Ivanyuk. The temperature dependence of thermal conductivity of some chemical elements. *Experimental Thermal and Fluid Science*, 4(3):289–300, May 1991.

[19] M. Asheghi, K. Kurabayashi, R. Kasnavi, and K. E. Goodson. Thermal conduction in doped single-crystal silicon films. *Journal of Applied Physics*, 91(8):5079–5088, March 2002.
